# Integrating socio-ecosystemic factors in One Health approaches: a scoping review in zoonotic disease research

**DOI:** 10.1016/j.onehlt.2025.101086

**Published:** 2025-05-24

**Authors:** Anthony Giacomini, Agnès Waret-Szkuta, Tephanie Sieng, Didier Raboisson, Guillaume Lhermie, Marisa Peyre, Hélène Guis

**Affiliations:** a UMR ASTRE, Université de Toulousel, ENVT, Toulouse, France; b Epidemiology & Public Health Unit, Institut Pasteur du Cambodge, Phnom Penh, Cambodia; c UMR ASTRE, Univ Montpellier, CIRAD, INRAE, Montpellier, France

**Keywords:** One Health, Socio-ecosystemic, Factors, Zoonoses, Emergence

## Abstract

It is widely accepted that zoonoses pose an ever-growing threat which should be tackled using a One Health approach. Like One Health, the socio-ecosystemic framework is a powerful concept that has been used to study complex systems. The aim of this work is to identify the socio-ecosystemic factors influencing zoonotic risk, while assessing and highlighting the importance of using this framework from a One Health perspective. To do so, we conducted a scoping review following the PRISMA-ScR guidelines on articles written in English, published between 1986 and 2023, and extracted from three scientific databases (PubMed®, Scopus® and Web of Science™) in January 2023 or identified by snowball sampling. Eligibility criteria were applied to select relevant scientific articles from various disciplines, related to the epidemiology of zoonotic agents in a context of epidemic risk. Among the 1661 articles extracted from the databases, to which must be added articles identified by snowball sampling, a total of 195 articles that underwent full-text analyses were included in our work. We identified 47 socio-ecosystemic factors influencing the risk of zoonotic emergence: 15 were categorized as ecological, 15 as economic, and 17 as social. The social factor “hunting, poaching and wildlife trafficking” was the most referenced, with 24 citations. Only 15 % of the studies were considered truly “interdisciplinary” because they integrated all three dimensions of the socio-ecosystem, and their publication dates show that the issue has only recently been taken up. These results provide a relevant synthesis of knowledge on the subject, in an original way that underlines the equal importance of these three dimensions. The corpus of articles is covered with a good saturation of references, ensuring the representativeness of the results. This work could contribute to a more holistic and effective consideration of the risk of zoonotic emergence, by emphasising the importance of interdisciplinarity.

## 1 Introduction

One Health is a conceptual frame for holistic and systemic thinking that meets the need to decompartmentalise the various fields of science concerned by complex public health challenges and implement transdisciplinary research to address them [1]. Among those challenges, the emergence of zoonotic diseases – diseases transmitted between human and non-human animal reservoirs – is a major threat to our societies, as the COVID-19 pandemic reminded us. These diseases represent 72 % of all emerging diseases of the past few decades, and their emergence are influenced by the social and economic features of our societies and the natural environment in which those societies thrive [[2], [3], [4], [5]]. Here, a (re)emergence was considered an event in which the actual incidence of a disease increased significantly in a given population in a given region over a given period of time compared with the usual epidemiological situation [6]. The notion of a socio-ecosystem, which acknowledges the complex interactions between ecosystems and human societies, meets the need to comprehend phenomena that have been difficult to analyse in a mono-disciplinary manner [7]. There is still a lack of uniformity in the definition of a socio-ecosystem across disciplines. Elinor Ostrom, who received the Nobel Prize in Economics in 2009, defines the socio-ecosystem as a framework for the economic study of ecosystem (i.e., an area providing a variety of ecosystem services) resilience when studying the governance of the commons [8]. Many definitions exist in the social sciences. In sociology, it can be defined as the context in which people are evolving in regard to evaluating knowledge or practices, for example, knowledge about fire management and wildfires in a specific community living in a given ecosystem (i.e., a biome with given biotic and abiotic characteristics) [9]. In ecology, for example, with respect to landscapes and vegetation floristic and structure, the socio-ecosystemic framework can be understood as a definition of the landscape and the environment that considers biophysical and anthropic variables [10]. In this study, we used the “socio-ecosystem” definition of the French National Research Institute for Agriculture, Food and Environment (INRAe), which best fits our study purpose, describing it as a conceptual framework of environmental dynamics at a local scale in which ecological and social–economic compartments are in perpetual and reciprocal interplay. Both compartments are made up of varied and complex structures and feature processes that provide socio-ecosystemic services [11]. With respect to diseases, we believe that those processes and structures can act as epidemiologic factors, as they are “events, characteristics, or other definable entities that have the potential to bring about a change in a health condition or other defined outcome” according to the definition of the United States National Library of Medicine (MeSH Unique ID: D015981).

In epidemiology, a substantial corpus of articles focuses on individual risk factors linked to exposure, infection or the severity of the infection. Many of them are studied separately in different fields: for example, in analytical epidemiology and ecology for environmental exposure factors, or in knowledge-attitude-practice (KAP) studies for socioeconomic or cultural exposure factors. These articles typically include socio-demographic characteristics (gender, age…) or socio-economic characteristics (income, employment…) and can also include environmental conditions (living in a rural or urban area, spending time near given environmental settings…). However, when focusing on the population level, we found it difficult to find articles in the literature that jointly integrate social, economic and environmental parameters to address the drivers of disease emergence. In this paper, we use the term “socio-ecosystemic factors” to refer to structural and systemic factors. We assume that the integration of socio-ecosystemic factors in One Health approaches is necessary to provide a complete picture. This is based on a clearly identified lack of cross-sectoral collaboration and cooperation in the joint collection and analysis of data [12,13]. We conducted a review to better identify these social, economic and ecological factors, published in the scientific literature, that influence the risk of zoonotic emergence. More precisely, a scoping review was conducted here, as it is intended to determine the coverage of a body of literature on a given topic and its focus but also to search for the key characteristics or factors related to a concept [14]. In addition, in this corpus of articles adopting a One Health approach, we assessed the proportion that considered the three dimensions of the socio-ecosystem, thus implementing an interdisciplinary methodology. Our study is one of the first necessary steps in synthesizing knowledge into an innovative, enlarged One Health lens of analysis. We have constructed an analytical framework that considers both the socio-ecosystemic study framework and the One Health approach to studying health problems (described in Supplementary Fig. A.1).

## 2 Methods

### 2.1 Definitions

The term “factors” was categorized into three dimensions: “economic” is linked to the production, allocation and consumption of resources and services; “social” refers to the sum of individuals, their interactions, and the emanating superstructures (e.g., political, religious, or juridical systems); and “ecological” is related to the natural environment in which animal and human populations live and their biotic and abiotic properties [[15], [16], [17], [18]]. The factors were labelled according to what they were perceived to have as their dominant dimension.

### 2.2 Scoping review protocol

**Fig. 1:**
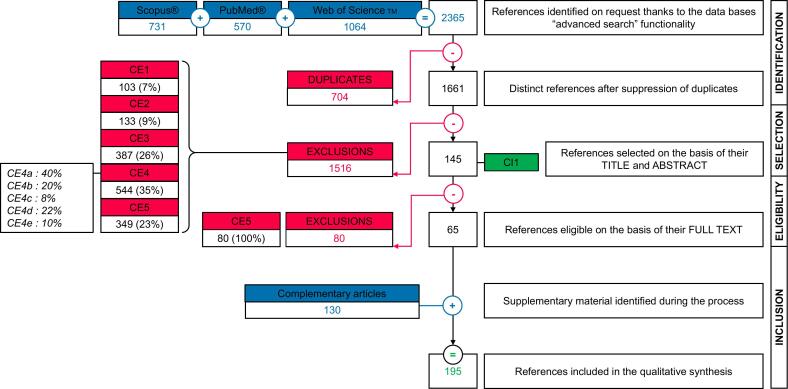
Summary flow chart of the scoping review: 1CE– Inadequate format. 2CE- Irrelevant field of research. 3CE– Absence of applied epidemiology. 4CE– Non-zoonotic agent with (A) Pollution and environmental quality degradation, (B) Ecological disruptions, (C) Non-infectious effects, (D) Antibiotic resistance, (E) Others. 5CE– Absence of relevant factors. CI1– Explicit presence of zoonotic risk.

We followed the instructions of the PRISMA-ScR method: an extension for prospecting scoping of the PRISMA method (Preferred Reporting Items for Systematic reviews and Meta-Analyses) [19]. All the methodological points were validated and then double-checked using the checklist of Joanna Briggs Institute (JBI) guidelines (Appendix C) [20]. A summary flow chart was constructed to visualize the process (Results, Fig. 1).

### 2.3 Eligibility criteria

Only articles in English were retained. The selected articles were published between January 1973 and January 2023.

The inclusion criterion used in the different selection phases was “Explicit presence of zoonotic risk factor”. The exclusion criteria were “Inadequate format”; “Irrelevant field of research”; “Absence of applied epidemiology” when there was no use of methods or tools derived from epidemiology (for example, fundamental bacteriologic study of a pathogen) or any implications at the population scale (for example, laboratory analytic test performance assessments); “Nonzoonotic agent” (for example, non-infectious health conditions or environmental pollution and degradation); and “Absence of relevant factors”, as only factors whose association with one or more zoonotic agents (circulating within a specific socio-environmental setting) was clearly described in the text were included.

### 2.4 Information sources

Three relevant generalist online scientific databases were used to retrieve digital or digitalized articles: PubMed® (1974–present), Scopus® (1979–present) and Web of Science™ (1990–present) [21]. The results were retrieved on January 16, 2023. No automation tools were used. A snowball sampling method was used: if a full-text article cited another relevant reference (i.e. meeting the same eligibility criteria), this reference was added to the list of complementary articles; this process is summarized in Supplementary Table A.3. The complete database, detailed step by step, is available in Appendix B.

### 2.5 Search strategy

To find relevant articles, a query using the “advanced research” functions of the search engine of each database was created. Additive arguments were defined using various lexical fields: one for the socio-ecosystemic framework (*ecosystem*, socio-ecosystems, “One Health”), one for the risk notion (“indicator”, risk factor, assessment, monitoring, participative study, survey), one for the zoonotic notion (epidem*, zoono*, “public health”) and one with thematic words related to ecological, economic or social features (social, societ*, sociolo*, politic*, cultural, religi*, anthropo*, ethno*, econo*, financial*, monetary, trade, ecolo*, environment*).

The asterisk (*) allowed for truncation: all words built on the common root preceding the asterisk were retained. Double quotation marks (“) indicate strict spelling: for “indicator“ because otherwise, the verb “indicate“ would also have been included in the query, and for “public health“ and “One Health“ to avoid inclusion of any occurrence of the word “health”.

The logical structure of the query included four arguments: argument (A) integrated the notion of One Health and social-ecosystem from the title and abstract; arguments (B) and (C) presented the notions of indicators, risk factors and zoonoses from the title or abstract; and argument (D) provided all the thematic vocabulary needed for the search in the entire text. In the following example, the letters (A) to (D) delimit the arguments and were not included as such in the query. For the two other databases, the structure of the query was the same, adapting the syntax of the logical operators to the respective specific “advanced search” modes. Thus, the PubMed® query was as follows:

(A) (ecosystem*[Title/Abstract] OR socio-ecosystem*[Title/Abstract] OR “One Health”[Title/Abstract])

(B) AND (“indicator”[Title/Abstract] OR risk factor[Title/Abstract] OR assessment[Title/Abstract] OR monitoring[Title/Abstract] OR participative study[Title/Abstract] OR survey[Title/Abstract])

(C) AND (epidem*[Title/Abstract] OR zoono*[Title/Abstract] OR “public health”[Title/Abstract])

(D) AND (societ* OR social OR sociolo* OR religi* OR anthropo* OR ethno* OR cultural* OR econo* OR financial OR trade OR monetary OR ecolo* OR environment* OR politic*)

### 2.6 Data extraction and analysis process

The selected records were exported from the databases into Microsoft® Excel® version 2301. Duplicates were manually deleted based on the title and authors and/or digital object identifier (DOI). For the total number of cleared records, missing DOIs were searched for in the article or on the page of the hosting site and were completed manually when possible. During the first selection phase (using the previously described eligibility criteria), the titles and abstracts of these papers were read, thus reducing the number of articles retained. From this reduced corpus, the articles were read in their entirety. The same criteria used during the first round were used for the second round.

As the articles were read through, the identified factors were entered into an Excel file developed for the purposes of the study. Each line corresponded to a given factor in a given study (one article could list several factors), with several variables documented in columns: name; brief description; references of the associated studies; study scale (global, international, national or local); study region (Central and West Africa, North Africa, South Africa, North America, Central and South America and the Caribbean, Southeast Asia, Central Asia and the Far East, South Asia and the Indian Ocean, Europe or Oceania); nature of the factor (ecological, social or economic); nature of the risk studied (only viral, bacterial, endoparasitic, ectoparasitic, mixed or nonconventional); associated pathogen(s) if named; and the categories of animal species concerned (companion or hobby animals, livestock, wildlife, synanthropic species or invertebrates (especially vectors)). The complete database, detailed step by step, is available in Appendix B.

### 2.7 Synthesis of the scoping phase: Categorization of identified factors

Following the completion of the scoping review process, redundant (same characteristics, same implications) or complementary (same characteristics, different implications) factors were identified and categorized under a synthetic name, and their descriptions were merged, along with their references. A rereading for reformulation and cleaning was then carried out.

### 2.8 Descriptive analysis

First, metadata from the articles were analysed to identify the main trends in terms of distribution, chronology, fields of research, interdisciplinarity and methodologies. The extracted factors were subsequently analysed to identify the main trends in terms of distribution, animal population, type of factor (its main socio-ecosystemic dimension), and pathogens. In both cases, regionalized comparisons were carried out by continent. To test whether or not there was a “COVID-19 effect”, we looked for changes in the distribution of article publication dates for the three main factors.

## 3 Results

### 3.1 Flow chart of the review process

Overall, 65 studies were included out of 2365 initially identified. A pool of 130 complementary studies was identified among the references cited in the 65, leading to a total of 195 studies included in the scoping review (Fig. 1).

### 3.2 Socio-ecosystemic factors

After categorization, a total of 47 unique factors were identified from the selected studies: 15 ecological, 15 economic and 17 social (Table 1, Table 2, Table 3, with full descriptions provided in Supplementary Table A.2). They were sorted by decreasing number of references and then by alphabetical order.

**Table 1:** Fifteen ecological factors influencing the risk of zoonotic emergence and their associated numbers of references.

Ecological factor	Number of references
Disasters and increases in the frequency and intensity of extreme climatic events linked to climate change	19 [[22], [23], [24], [25], [26], [27], [28], [29], [30], [31], [32], [33], [34], [35], [36], [37], [38], [39], [40]]
Fragmentation of the habitat by human infrastructures and anthropization (urban and agrarian landscapes, transport and energy infrastructure developments)	17 [22,[41], [42], [43], [44], [45], [46], [47], [48], [49], [50], [51], [52], [53], [54], [55], [56]]
Presence of intensive agrosystems (or those undergoing intensification) and monocultures, land expansion and agricultural infrastructures	17 [22,25,41,42,46,[57], [58], [59], [60], [61], [62], [63], [64], [65], [66], [67], [68]]
Deforestation	15 [42,45,57,[69], [70], [71], [72], [73], [74], [75], [76], [77], [78], [79], [80]]
Urbanization	11 [22,25,42,77,[81], [82], [83], [84], [85], [86], [87]]
Disturbance of environmental equilibria, linked to climate change	9 [26,73,77,[88], [89], [90], [91], [92], [93]]
Multiplicity of farming systems and practices (livestock and/or pastoralism) and mixing of animals with each other and with wildlife in pastures and around watering holes	4 [26,[94], [95], [96]]
Biodiversity collapse	3 [[97], [98], [99]]
Farmland abandoned to forest and scrubland	3 [57,100,101]
Introduction of non-native species, especially invasive alien species (IAS)	3 [98,102,103]
Large populations of stray, nonmedicalized domestic carnivores	3 [69,104,105]
Regular use of the forest for recreational purposes	2 [57,106]
Weak urban greening and poor management of green spaces	2 [41,107]
Conflicts of use between humans and wildlife	1 [73]
Ecosystem services and resource depletion	1 [74]

**Table 2:** Fifteen economic factors influencing the risk of zoonotic emergence and their associated numbers of references.

Economic factor	Number of references
Hunting, poaching and wildlife trafficking	24 [22,73,82,[108], [109], [110], [111], [112], [113], [114], [115], [116], [117], [118], [119], [120], [121], [122], [123], [124], [125], [126], [127], [128]]
Economic recession, poverty, austerity and wealth inequality	13 [41,108,[129], [130], [131], [132], [133], [134], [135], [136], [137], [138], [139]]
Weakness of health infrastructures due to a lack of material and human resources	13 [129,[131], [132], [133], [134], [135], [136], [137], [138],[140], [141], [142], [143]]
Presence of extractive industries: mining or cave-related activities, political actions favouring mining in indigenous territories	12 [22,42,[144], [145], [146], [147], [148], [149], [150], [151], [152], [153]]
Existence of a bushmeat production chain	6 [77,92,110,[154], [155], [156]]
Lack of resources to build and maintain sanitation and waste management infrastructures	6 [41,91,96,[157], [158], [159]]
Population movements through migration or nomadic lifestyles	6 [25,42,77,[160], [161], [162]]
Globalization of trade, connectivity of cities and countries	4 [74,77,140,163]
Professional activities involving exposure to animals by handling animals or soiled work utensils	4 [69,110,164,165]
Wet markets	3 [69,154,166]
Industrialization of the agri-food sector	2 [108,167]
Slaughter or discount sale of sick animals or their meat	2 [69,168]
Cost of specific therapies too high for hospital systems in developing or underdeveloped countries (here, postexposure prophylaxis for rabies)	1 [104]
Differential funding for zoonosis management depending on the disease (with a particular preference for malaria)	1 [25]
Illegal imports of meat or uncontrolled meat	1 [102]

**Table 3:** Seventeen social factors influencing the risk of zoonotic emergence and their associated numbers of references.

Social factor	Number of references
Lack of public knowledge about zoonotic risk factors due to a lack of communication and awareness-raising or misinformation	21 [129,[131], [132], [133], [134], [135], [136], [137], [138],[169], [170], [171], [172], [173], [174], [175], [176], [177], [178], [179], [180]]
Belonging to a neglected or stigmatized minority	17 [22,59,108,169,172,178,[181], [182], [183], [184], [185], [186], [187], [188], [189], [190], [191]]
Poor governance due to lack of political will, lack of resources, degraded or inadequate public health measures	15 [104,129,[131], [132], [133], [134], [135], [136], [137], [138],^142^,^143^,^169^,^178^,^192^]
War, famine, political instability and conflict	14 [96,108,129,[131], [132], [133], [134], [135], [136], [137], [138], [139],^193^,^194^]
Consumption of contaminated or high-risk food products in daily life	12 [69,82,92,96,108,164,169,[195], [196], [197], [198], [199]]
Declining public confidence in government public health interventions, especially vaccination	10 [129,[131], [132], [133], [134], [135], [136], [137], [138],^142^]
Ecotourism and international travel activities	7 [82,108,140,[200], [201], [202], [203]]
Lack of knowledge and training among health care professionals	6 [25,169,175,177,181,204]
Colonial practices and illegal activities in indigenous territories	4 [22,[205], [206], [207]]
Disparities in knowledge and communication problems between professionals	4 [169,172,177,208]
Pet ownership	4 [108,199,209,210]
Poor butchery practices and management of butchery waste and carcasses	3 [69,154,211]
Religious and traditional beliefs involving contact with animals or the consumption of contaminated or risky food products within a ritual or traditional event	3 [110,141,158]
Societal-level gender bias in the access, production and dissemination of information regarding risky practices and the distribution of risky tasks	2 [212,213]
Livestock in free circulation in the residential area	2 [158,214]
Negative cultural influence on care-seeking	1 [141]
Political and socioeconomic organizations of society that limit the implementation of restrictive, individual, preventive measures	1 [140]

### 3.3 Descriptive analysis of the studies

**Fig. 2:**
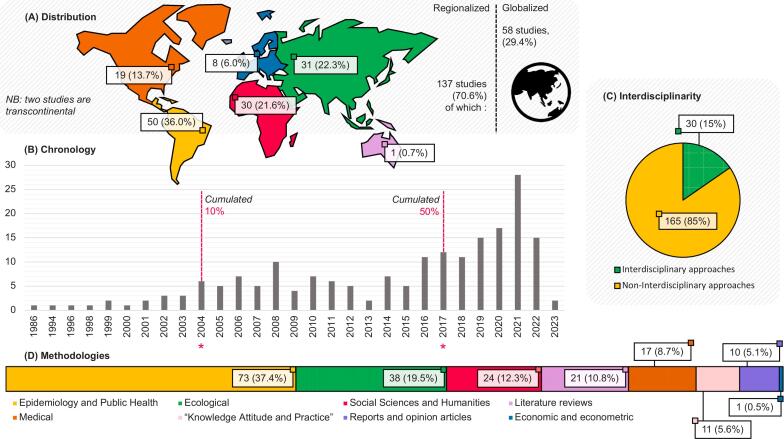
Descriptive characteristics of the studies: (A) Continental distribution of the articles normalized to 100 %, one regionalized study can exemplify several regions. (B) Chronologic distribution of the studies from 1986 to 2023. Asterisks (*) indicate the cumulative 10 % and cumulative 50 % in 2004 and 2017, respectively. (C) Number of studies jointly analysing ecological, economic and social data. (D) Methodologies used.

Among the studies included in the review, more than two-thirds focused on at least one specific region: Central and South America and the Caribbean was the first region in terms of the number of studies (25.5 %), with 51 times more articles than the last region, Oceania (0.5 %). This body of literature covers the last forty years of research (1986–2023), with half of the papers written from 2017 onwards. Only 15 % of the studies were considered truly “interdisciplinary” because they conjointly exploited and analysed economic and environmental and social data. These studies ranged from 2004 to 2022, with more than 50 % published in 2021 and 2022. Epidemiologic methodologies dominate among the eleven categories identified (37.3 %), with economic and econometric methodologies at the lower end of the ranking (Fig. 2).

### 3.4 Descriptive analysis of the factors

The mean number of references identified per factor was 7, regardless of the type of factor, with standard deviations of 7 for the ecological factor and 6 for the economic and social factors, and medians of 3, 4 and 4, respectively. Approximately 13 ecological, 12 economic and 15 social factors were cited in several articles (85.1 % of the total), whereas only 2 ecological, 3 economic, and 2 social factors were referenced in only one article (14.9 % of the total). The most referenced factors per dimension were “Disasters and increase in frequency and intensity of extreme climatic events linked to climate change” referenced 19 times, “Hunting, poaching and wildlife trafficking” referenced 24 times, and “Lack of public knowledge about zoonotic risk factors, by lack of communication and awareness-raising, or by misinformation” referenced 21 times (Table 1, Table 2, Table 3). For these three factors, basic descriptive analysis did not reveal any “COVID-19 effect” on the publication rates of the related studies.

**Fig. 3:**
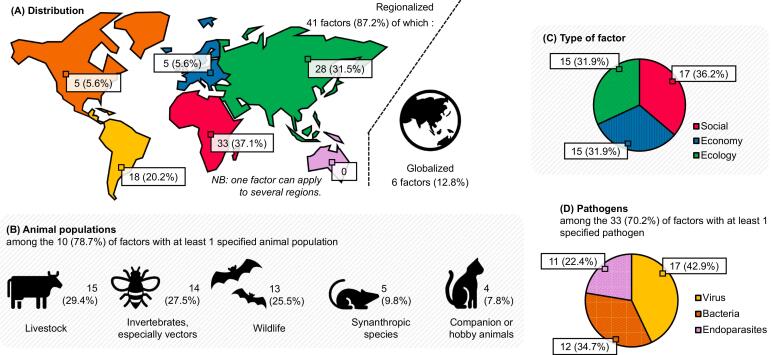
Descriptive characteristics of the factors: (A) Continental distribution of the factors, one regionalized factor can apply in several regions, and regionalized percentages are set to 100 %. (B) Animal populations concerned. (C) Type of factor. (D) Type of pathogens.

The vast majority of factors concerned at least one specific region, including Africa at the top of the rankings (33, i.e., 37.1 %), Asia (28, i.e., 31.5 %) and South and Central America and Caribbean (18, i.e., 20.2 %). The interface between livestock (29.4 %) and wildlife (25.5 %), including vectors (27.5 %), accounted for most of the focus, whereas other animal populations were less prominent. Social factors were slightly more represented than economic and ecological factors were, both of which were equally represented. More than two-thirds of the factors mentioned at least one specific pathogen, the majority of which were viruses, followed by bacteria, and finally, endoparasites (Fig. 3).

The 33 factors that mention at least one specific pathogen (70.2 %) could refer to species, genera or families. The most frequently cited viruses were Arboviruses (six times). The most frequently cited bacteria were those of the Brucella genus (six times), particularly *Brucella abortis*. The most frequently cited parasites were *Taenia solium* and *Plasmodium* sp. (three times respectively), in particular *Plasmodium knowlesi*. These results are summarized in Table 4.

**Table 4:** Pathogen types and names for the 33 factors associated with at least one specified pathogen.

Type of pathogen	Name	Count ⁎
Virus	Arboviruses (studied as a whole)	6
Virus	Nipah Virus (NiV)	3
Virus	Rabies Virus (RV)	3
Virus	Severe Acute Respiratory Syndrome Coronavirus 2 (SARS-CoV-2)	3
Virus	Influenza A Virus	2
Virus	*Hantaviridae*	2
Virus	Tick-Borne Encephalitis Virus (TBEV)	2
Virus	Crimean–Congo Haemorrhagic Fever Virus (CCHFV)	1
Virus	Chikungunya Virus (CHIKV)	1
Virus	Dengue Virus (DENV)	1
Virus	Kyasanur Forest Disease Virus (KFDV)	1
Virus	Rift Valley Fever Virus (RVFV)	1
Virus	West Nile Virus (WNV)	1
Virus	Zika Virus (ZKV)	1
Bacteria	*Brucella* spp., of which 1 *B. abortis*	6
Bacteria	*Leptospira* spp.	5
Bacteria	*Bacillus anthracis*	3
Bacteria	*Campylobacter* spp.	2
Bacteria	*Vibrio* spp.	2
Bacteria	*Enterocytozoon bieneusi*	1
Bacteria	*Francisella tularensis*	1
Bacteria	*Mycobacterium tuberculosis*	1
Bacteria	*Mycobacterium ulcerans*	1
Bacteria	*Rickettsia tsutsugamushi*	1
Bacteria	*Salmonella* spp.	1
Endoparasite	*Taenia solium*	3
Endoparasite	*Plasmodium* spp., of which 1 *P. knowlesi*	3
Endoparasite	*Trichinella spiralis*	2
Endoparasite	*Trypanosoma* spp., of which 1 *T. cruzi*	2
Endoparasite	*Leishmania* spp.	1

⁎ : One factor could be related to several pathogens, for a total count of 63 mentions of pathogens.

## 4 Discussion

This scoping review led to the identification of 47 factors influencing zoonotic emergence: 15 ecological, 15 economic, and 17 social factors (Table 1, Table 2, Table 3, full descriptions provided in Supplementary Table A.2). The type and the number of references of the 47 factors identified were balanced among the three dimensions of the socio-ecosystem. There is a clear disciplinary imbalance in the methodologies used, coupled with the fact that only 15 % of studies actually linked social, economic and ecological data simultaneously in their analysis. However, fully integrated methodologies including the three dimensions of the socio-ecosystem are increasingly being implemented, with a marked upward trend since 2021. The factors “hunting, poaching and wildlife trafficking”, “disasters and increases in the frequency and intensity of extreme climatic events linked to climate change” and “lack of public knowledge about zoonotic risk factors due to a lack of communication and awareness-raising or misinformation” were the most referenced factors.

Our study successfully demonstrated that the number of risk factors was equivalent across the three dimensions of the socio-ecosystem. In addition to providing a relevant synthesis of the data in the literature, we assessed the proportion of the corpus that actually incorporated these three dimensions into their analysis. Although this proportion is low (Fig. 2), the very recent acceleration in the rate of publication of these studies is encouraging, as it shows that these analyses are not only feasible, but that their importance is understood. This confirms the need to continue to call for a conception of epidemic risk prevention that is both One Health and socio-ecosystemic, in other words truly systemic. This conception encompasses both a joint consideration of the affected human and non-human populations in their environment, and an interdisciplinary appreciation of the complexity of that environment.

In interpreting the results, some conceptual considerations must be discussed. First and foremost, as a first scoping study, the aim of this work was neither to quantify the effects nor to assign a degree of importance to each of the 47 factors identified. Rather, the aim was to produce an interdisciplinary synthesis of the factors identified in the literature and to compare their distribution across the three dimensions of the socio-ecosystem. Therefore, this work alone is not a functional risk assessment tool (for instance, it could not be used as a checklist in a monitoring system yet). Although this scoping review was carried out as a single-operator screening for practical reasons, it provides a strong basis for a systematic review and elicitation of expert opinion to assess the relative importance and priority of the various factors identified here in each dimension [14,215]. To a lesser extent, we also need to discuss the notion of risk factor. The Terrestrial Animal Health Code of the World Organization for Animal Health (WOAH) defines a risk as “the likelihood of the occurrence and the likely magnitude of the biological and economic consequences of an adverse event or effect to animal or human health” [216]. Among the several meanings of the word “risk”, this definition is a concept of risk applied to disease surveillance in veterinary medicine and veterinary public health [217]. We did not refer to the socio-ecosystem factors we identified as “risk factors” because they were not necessarily considered from a monitoring and control perspective in the original article. This would have been all the more confusing as we only scoped the literature and did not quantify the impact of each factor in all circumstances. This is why these items were described in this paper as “factors influencing zoonotic risk”, pending further investigation.

Some methodological limitations also need to be acknowledged when interpreting the results. Firstly, the choice of query arguments significantly influenced search results. For example, including the concepts of “One Health” or “socio-ecosystem” in the abstract is a very restrictive choice. Removing this constraint increases the number of identified articles from 2365 to 1,502,401. This choice allowed us to narrow the selection of studies to our working framework (Supplementary Fig. A.1) and to exclude as many studies as possible that made only a brief mention of One Health or the socio-ecosystem at the end of the discussion. While the query was restrictive, the inclusion of 130 complementary articles by the snowball sampling limited the risk of not identifying critical factors. Such a large number of complementary studies confirms that more studies could have been potentially included. Still, the fact that 85.1 % of the factors were cited multiple times (with a mean number of citations of 7) suggests that we have reached sufficient saturation of data from the literature. Secondly, this snowball sampling process may have led to the overrepresentation of articles from two reviews from “Central and South America and the Caribbean” [157,218]. However, the synthesis of the “raw” extraction factors into 47 multi-regional factors has corrected this bias, relegating “Central America, South America and the Caribbean” from first place in terms of number of articles to third place in terms of number of factors at this stage. The risk zones indicated for each factor (Fig. 3, full descriptions provided in Supplementary Table A.2) are limited to the data in the literature; however, it is reasonable to think that some factors may in fact apply beyond these zones. The geographical data associated with each factor should therefore be regarded with some caution.

In terms of concrete recommendations and actions, more work needs to be done to hierarchize and weight each factor. This could be done using a mixed method: for example, a systematic review of a large number of articles combined with a meta-analysis to estimate the strength of the effects quantitatively, and participatory methods involving the populations concerned to collect qualitative data that will allow us to assess the relative importance and perception of the factors and help to identify new, unexplored factors. Then, from a public health point of view, to allow for tangible actions on those factors, they must be kept under surveillance. To do so, we need to define indicators to monitor. Indeed, disease surveillance is a major step in the building of solid foundations for global public health, but while the need for surveillance is increasing, entire areas of research – such as economic evaluation, for example – are still poorly included in surveillance systems, while the derived benefits outweigh the cost of implementation [219]. Expanding the co-design of One Health surveillance systems to include indicators of the socio-ecosystemic framework would provide critical data to ensure relevant and impactful implementation of health policies in the field. A study on this topic is currently being carried out by an expert working group of the PREZODE initiative and the WHO, to provide evidence for decision making, based on indicators to monitor risk reduction [authors].

## 5 Conclusions

In her 1962 best-selling book, Silent Spring, environmentalist Rachel Carson wrote: “In nature, nothing exists alone” [220]. The enormous impact of this book on society at the time can be explained, in part, by the pioneering spirit it contained: considering humans, animals and the environment as a dense and complex web, where the consequences of a health crisis can spread in unforeseeable ways. Socio-ecosystemic and One Health approaches are a tiny but necessary step towards a more holistic conception of major public health issues, such as zoonotic emergences, thus illustrating that in every socio-ecosystem, nothing exists alone.

## CRediT authorship contribution statement

**Anthony Giacomini:** Writing – original draft, Visualization, Methodology, Formal analysis, Conceptualization. **Agnès Waret-Szkuta:** Writing – review & editing, Methodology, Conceptualization. **Tephanie Sieng:** Writing – review & editing, Validation, Supervision, Resources, Project administration. **Didier Raboisson:** Writing – review & editing. **Guillaume Lhermie:** Writing – review & editing, Conceptualization. **Marisa Peyre:** Writing – review & editing, Validation, Supervision, Resources, Project administration, Methodology, Conceptualization. **Hélène Guis:** Writing – review & editing, Validation, Supervision, Resources, Project administration, Methodology.

## Funding

This work was accomplished within the PREACTS AfriCam project, funded by the 10.13039/501100011061French Development Agency (AFD), under the PREZODE International Initiative. It was supported by 10.13039/501100007204CIRAD, the international mobility grant scheme for students offered by the Occitanie Region of France (n°23006423), and the 2023 international internship grant scheme for students offered by the France Veterinary International service of the National School of Veterinary Services (ENSV-FVI).

The award of student grants had no impact on the study design, data collection, data analysis, data interpretation, or writing of the report.

## Declaration of competing interest

The authors declare the following financial interests/personal relationships which may be considered as potential competing interests:

Anthony Giacomini reports financial support was provided by VetAgro Sup. Anthony Giacomini reports financial support was provided by Occitanie Region. If there are other authors, they declare that they have no known competing financial interests or personal relationships that could have appeared to influence the work reported in this paper.

## Data Availability

The complete list of all the articles at every step of the review and all the extracted information used in this article can be found in the supplementary materials of the publication.

## Appendix A Supplementary data

Supplementary material 1 Image 1

Supplementary material 2 Image 2

Supplementary material 3 Image 3
