# Supplementary material for: Integrating socio-ecosystemic factors in One Health approaches: a scoping review in zoonotic disease research
Source: One Health. 2025 May 24;20:101086. doi: 10.1016/j.onehlt.2025.101086 (PMC12158535; doi:10.1016/j.onehlt.2025.101086)
Supplement: Supplementary file 1 — Supplementary material 1 [file mmc1.pdf]

Integrating socio-ecosystemic factors in One Health approaches: a scoping review in zoonotic disease research. Supplementary Material

Anthony Giacomini*, Agnès Waret-Szkuta, Tephanie Sieng, Didier Raboisson, Guillaume Lhermie, Marisa Peyre, Hélène Guis

Figure A.1: Schematic representation of our working framework...p.2
Table A.2: Full description of risk factors and the associated studies identifiers...........................p.3
Table A.3: Articles included and their links..p.14

Figure A.1 - Schematic representation of our working framework.

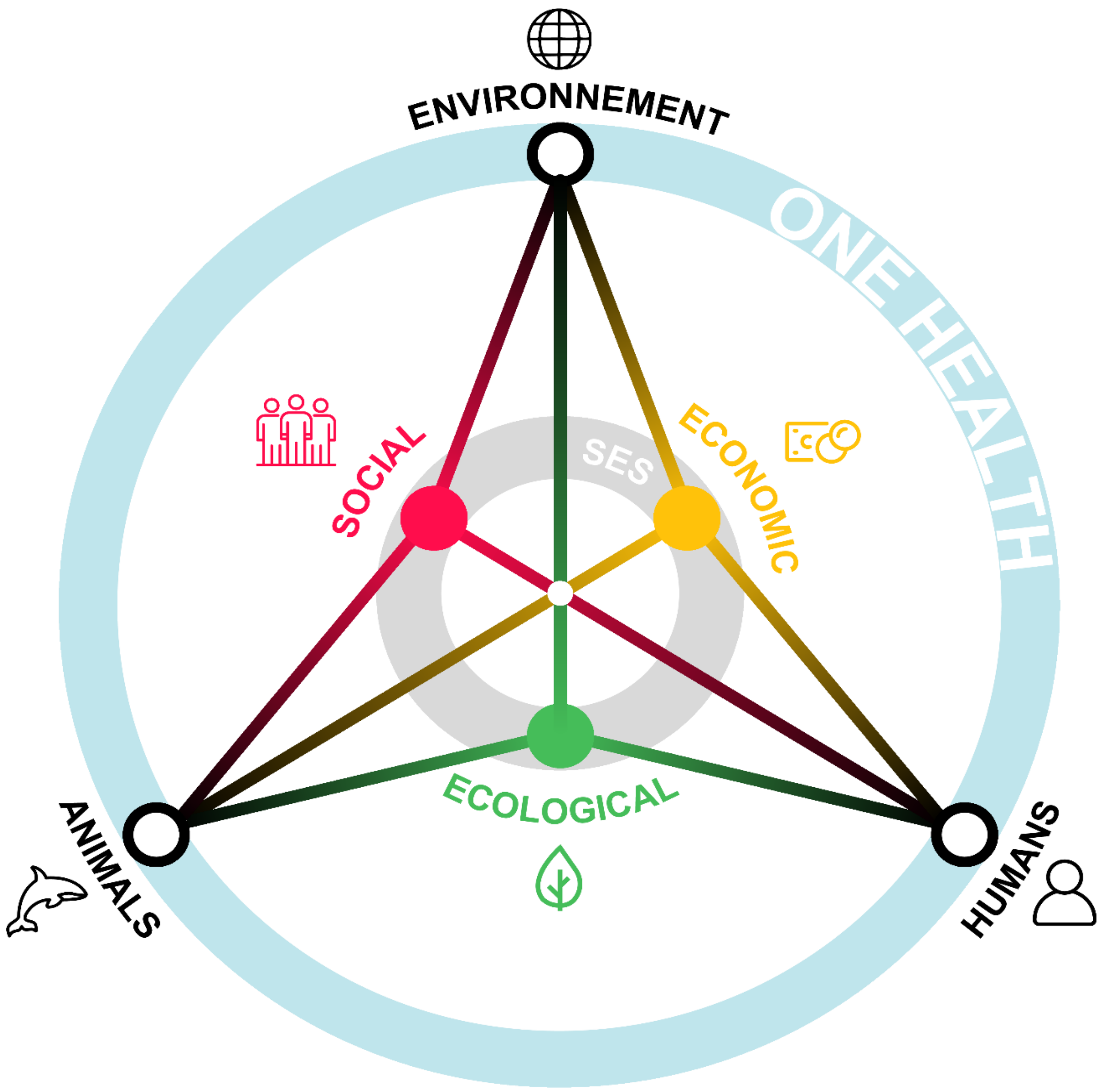

The outer circle corresponds to the components of the One Health concept, the inner circle to the components of the socio-ecosystemic framework (SES), and the lines represent all the interactions that then need to be considered in the study of a public health problem.

Table A.2 - Full description of risk factors and the associated studies identifiers

ID		FACTORS	DESCRIPTIONS
ECOLOGY	FA020	Disasters and increases in the frequency and intensity of extreme climatic events linked to climate change	*Natural disasters and extreme climatic events undermine human and animal populations, public health systems and food safety... Climate change is leading to an increase in the frequency and intensity of some of these extreme events, notably flooding in tropical zones, thus favoring the development and spread of certain pathogens or influencing the population dynamics of generalist and synanthropic hosts such as rodents. Climatic disturbances such as particulate pollution linked to the combustion of fossil fuels and the melting of permafrost increase the risk. Area specified in the studies are found throughout the world, but particularly in Africa, Central Asia and the Far East, especially in the case of bacterial pathogens such as Leptospira spp. and arboviral pathogens.*
		**19 ARTICLES**	
		C021, C088, C089, C090, C091, C092, C093, C094, C095, C096, C097, C098, C099, C100, C101, A028, A037, A051, A064	
ECOLOGY	FA037	Fragmentation of the habitat by human infrastructures and anthropization (urban and agrarian landscapes, transport and energy infrastructure developments)	*Road building, logging, urban, agricultural or energy infrastructure development, etc., fragment the environment into closed patches, confining animal populations to small areas where emergence is facilitated. This habitat degradation has harmful effects on the nutrition and immunity of animals present in high densities in these areas, increasing the zoonotic risk. Changes in the population dynamics of vectors and their predators can be observed in these fragmented territories. Areas identifed in the studies are found throughout the world, but particularly in Central and South America, the Caribbean and Southeast Asia, especially in the case of endoparasitic pathogens such as Plasmodium knowlesi.*
		**17 ARTICLES**	
		A023, A024, C020, C054, C059, C060, C061, C062, C063, C064, C065, C066, C067, C068, A028, A034, A043	
ECOLOGY	FA034	Presence of intensive agrosystems (or those undergoing intensification) and monocultures, land expansion and agricultural infrastructures	*The presence of agro-ecosystems, through the conversion of preserved areas into farmland or the intensification and creation of monoculture areas, acts as a "biodiversity filter", favouring the expansion of a limited number of species of small generalist and synanthropic vertebrates (generally rodents) which often make very good hosts for diseases with zoonotic potential and which then interact with livestock and humans. Irrigation and the presence of irrigation structures can lead to very rapid growth in vector populations. Areas identifed in the studies are found throughout the world, but particularly in Central and South America and the Caribbean, especially in the case of arboviral pathogens such as East Nile virus or Nipah.*
		**17 ARTICLES**	
		C054, C055, A022, A023, A024, A028, A036, C069, C070, C071, C072, C073, C074, C075, C076, C077, A064	

ECOLOGY

Code	Factor	Description
**FA004**	Deforestation	*Deforestation increases the frequency of contact between humans, vectors, wild and domestic animals, both through the activity itself and its consequences. The resulting reduction in biodiversity alters population dynamics, often in favor of generalist species, vectors and parasites. High species richness in small mammals in areas of low deforestation and low species richness in small mammals in areas of high deforestation, are risk factors. Increases in vector size without changes in abundance have also been reported. Deforestation can give way to scrubland, favorable to generalist synanthropic host species and vectors. Sometimes, reforestation of deforested areas can also be a risk factor. Areas identifed in the studies can be found all over the world, but particularly in Central and South America, the Caribbean and throughout Asia, particularly in the case of bacterial pathogens such as Rickettsia tsutsugamushi, endoparasitic pathogens such as Leishmania spp. and Plasmodium spp. and viral pathogens such as Nipah, Hantaviridae and KFDV.*

15 ARTICLES

A001	A006	C008	A010	C019	C020	A022	C053	A024	A031	A035	A036	A039	A040	A045

Code	Factor	Description
**FA033**	Urbanization	*Massive urbanization combined with a sharp rise in human populations is encroaching on wildlife habitat, increasing the risk. Anthropized and densely populated ecosystems provide vectors with an abundant choice of hosts and oviposition sites, which is a risk factor. Areas identifed are found throughout the world, but particularly in Central and South America and the Caribbean, particularly in the case of bacterial pathogens such as Mycobacterium ulcerans and Leptospira sp. or arboviral pathogens.*

11 ARTICLES

C046	A024	A028	C117	C118	C119	C120	C121	A045	A058	A064

Code	Factor	Description
**FA021**	Disturbance of environmental equilibria, linked to climate change	*Climate change affects both the natural and social environments, modifying host/pathogen/environment dynamics. First example: rising temperatures linked to climate change favor the development of leptospires. Another example: the warming of Arctic zones allows the rapid progression of ectoparasites and vectors in naïve populations. Climate change in northern environments, and in particular the melting of permafrost, poses a risk in terms of the release of zoonotic bacterial and viral pathogens. A third example: warming surface waters and changes in salinity linked to climate change favor the development of Vibrio spp. A final example: climate disruption influences rodent populations, with periods of heavy rain and heat, for example, followed by periods of high rodent prolificacy. Areas identifed can be found throughout the world, but particularly in Africa, the Americas, South Asia and the Indian Ocean, particularly in the case of bacterial pathogens such as Leptospira spp. and Vibrio spp. or viral pathogens such as Hantaviridae.*

9 ARTICLES

C021	A020	A033	A035	A045	A047	A052	A062	A063

Category	Code	Factor	Description	Articles	Article IDs
ECOLOGY	FA009	Multiplicity of farming systems and practices (livestock and/or pastoralism) and mixing of animals with each other and with wildlife in pastures and around watering holes	*Practices involving the sharing of grazing land between different livestock herds of potentially different species (particularly cattle and sheep), or the grouping of animals around residual watering points in dry periods, increase the risk. The regions specified are Central and West Africa and South Asia and the Indian Ocean, particularly in the case of bacterial pathogens such as Brucella spp. or Leptospira spp.*	**4 ARTICLES**	A003, A012, C021, A025
ECOLOGY	FA045	Biodiversity collapse	*Biodiversity loss favors the development of certain generalist zoonotic pathogens, and stresses animal populations, making them more susceptible to infection. For example: the collapse of large wild carnivore populations increases their susceptibility to pathogens and their prevalence in populations, with subsequent communications to domestic carnivores. Areas specified are found worldwide, but particularly in Central and South America and the Caribbean, especially in the case of endoparasitic pathogens such as Plasmodium spp.*	**3 ARTICLES**	A048, A057, A066
ECOLOGY	FA035	Farmland abandoned to forest and scrubland	*The abandonment of farmland in favor of woodland or scrubland, particularly favorable to ticks, favors the emergence of bacterial diseases such as Lyme disease caused by Borrelia burgdorferi, or viral diseases such as TBEV. The whole world, without any particular distinction, is concerned.*	**3 ARTICLES**	C056, C057, A022
ECOLOGY	FA013	Introduction of nonnative species, especially invasive alien species (IAS)	*Invasive alien species (IAS), in 60% of cases imported voluntarily, can carry zoonotic parasites and pathogens that are new to a given area, and thus present a risk. The introduction of non-native host species (often accompanied by their pathogens) through globalization or climate change also presents a risk of introduction. Areas specified are found throughout the world, but particularly in Central Asia, the Far East and Europe, especially in the case of endoparasitic pathogens such as Trichinella spiralis.*	**3 ARTICLES**	A008, A056, A057
ECOLOGY	FA007	Large populations of stray, nonmedicalized domestic carnivores	*Maintaining large populations of stray, unvaccinated domestic carnivores, particularly in areas where the rabies virus is endemic, presents a risk. The regions specified are Southeast Asia, South Asia and the Indian Ocean.*	**3 ARTICLES**	A001, C007, A004
ECOLOGY	FA036	Regular use of the forest for recreational purposes	*Increased use of forests for mushroom and fruit picking by local populations increases contact with ticks. The main region specified is Europe, particularly in the case of viral pathogens such as TBEV.*	**2 ARTICLES**	C058, A022

Domain	Code	Factor	Description	Number of articles	Articles
ECOLOGY	FA038	Weak urban greening and poor management of green spaces	*While the "thoughtful greening" of urban areas reduces zoonotic risk by influencing habitat quality, "opportunistic greening", i.e. the creation of unmaintained vacant green spaces, combined with poor waste management in these green spaces, leads to the proliferation of certain pathogens. The regions specified are the Americas, particularly in the case of bacterial pathogens such as Leptospira spp. or endoparasitic agents such as Trypanosoma cruzi.*	**2 ARTICLES**	A023, A049
ECOLOGY	FA042	Conflicts of use between humans and wildlife	*Conflicts between humans and animals that ravage crops or are predators increase contact, hunting, poaching and consumption of these animals. The main region specified is South Asia and the Indian Ocean.*	**1 ARTICLE**	A035
ECOLOGY	FA043	Ecosystem services and resource depletion	*The depletion of natural resource reserves increases the pressure on alternative resources, which are generally more at risk. The whole world, without distinction, is concerned.*	**1 ARTICLE**	A036
ECONOMIC	FA030	Hunting, poaching and wildlife trafficking	*Hunting and poaching increase the risk of exposure to zoonotic diseases. Illegal wildlife trafficking has three main causes, in order: travel, the pet trade, and the bushmeat trade. This traffic is a zoonotic risk factor. Areas specified are found throughout the world, but particularly in Central and West Africa, Central and South America and the Caribbean.*	**24 ARTICLES**	A019, C044, C046, A028, C102, C103, C104, C105, C106, C107, C108, C109, C110, C111, C112, C113, C114, C115, C116, A030, A035, A046, A060, A061
ECONOMIC	FA015	Economic recession, poverty, austerity and wealth inequality	*Periods of economic recession and poverty are generally followed by epidemics, and vice versa (poor living conditions encourage infection, epidemics weaken the economy). Poverty and social inequality are the main economic drivers of the distribution of infectious disease morbidity and mortality. An inverse correlation between GDP and the incidence of Brucella spp. has been specified. Areas specified are found worldwide, but particularly in South and East Africa, Central and South America and the Caribbean, especially in the case of bacterial pathogens such as Brucella spp. or endoparasitic agents such as Trypanosoma spp.*	**13 ARTICLES**	A009, C010, C011, C012, C013, C014, C015, C017, C018, A019, C051, A023, A042

Category	Factor	Description	Explanation
ECONOMIC	FA019	Weakness of health infrastructures due to a lack of material and human resources	*The lack of resources and infrastructure in public health systems influences the appearance and evolution of epidemics: the lack of personnel, equipment and knowledge for prevention, detection and management... all present a risk. Areas specified are found throughout the world, but particularly in Africa and South-East Asia.*
		**13 ARTICLES**	
		A009, C009, C010, C011, C012, C013, C014, C015, C016, C017, C018, A050, A053	
	FA039	Presence of extractive industries: mining or cave-related activities, political actions favouring mining in indigenous territories	*Mining, particularly of gold, encourages the development of vectors and releases mercury (among other pollutants) into the environment, which has an immunosuppressive effect. Mining, particularly of gold, and associated practices, as well as political measures facilitating such extraction in indigenous territories, increase the risk for these populations. The whole world, without distinction, is concerned.*
		**12 ARTICLES**	
		A024, A028, C081, C082, C083, C084, C085, C086, C087, C128, C129, C130	
	FA012	Existence of a bushmeat production chain	*Hunting, capturing, transporting, sometimes farming and selling animals of different wild species represents a risk. Hygiene and management conditions for animals and meat are not optimal in these sectors. Flying foxes are one of the species most at risk. Intensive farming of wild animals is a prime location for the transmission of zoonotic pathogens. Areas specified are found throughout the world, but particularly in Central and West Africa and South-East Asia, especially in the case of viral pathogens such as COVID-19.*
		**6 ARTICLES**	
		A005, A007, A016, A045, A046, A062	
	FA025	Lack of resources to build and maintain sanitation and waste management infrastructures	*The lack of resources for sanitation and waste management infrastructures and their maintenance, combined with the habit of this lack, encourages, for example, the practice of outdoor defecation or exposure to sewage, which favors parasitic cycles. Exposure to domestic waste and sewage is a good indicator of habitat quality and exposure to vectors and competent hosts, such as rodents. Areas specified are found worldwide, but particularly in South and East Africa, Central and South America and the Caribbean, and Southeast Asia, notably in the case of bacterial pathogens such as Brucella spp. and Vibrio spp. or endoparasitic agents such as Taenia solium or viral agents such as Dengue, Chikungunya and Zika.*
		**6 ARTICLES**	
		A015, A023, A025, A026, A047, A054	

ECONOMIC	FA040	Population movements through migration or nomadic lifestyles	*The movement of animals and humans, through migration, travel, trade, nomadism, etc., encourages encounters with pathogens, and thus favors their emergence. The nomadic lifestyle is a fairly unique interface between livestock and humans, fostering zoonotic risk. Areas specified are found throughout the world, but particularly in Central and South America, the Caribbean, Central Asia and the Far East, particularly in the case of bacterial pathogens such as Mycobacterium tuberculosis or arboviruses.*
		**6 ARTICLES**	
		A024, A027, A041, A045, A059, A064	
	FA044	Globalization of trade, connectivity of cities and countries	*The transport of humans or livestock between different geographical areas favors the encounter between a zoonotic pathogen and its host. Numerous imports of goods and services, trade and uncontrolled livestock trade between countries are risk factors. Areas specified can be found all over the world, but particularly throughout Africa, especially in the case of viral pathogens such as Rift Valley Fever.*
		**4 ARTICLES**	
		A036, A045, A050, A065	
	FA002	Professional activities involving exposure to animals by handling animals or soiled work utensils	*Farm workers, inseminators, slaughterhouse workers, or animal practitioners... i.e. workers who clean utensils and feces, handle animals and feed them... are particularly at risk. The regions specified are Central and West Africa, Southeast Asia and Europe, particularly in the case of bacterial pathogens such as Brucella spp. and Campylobacter spp. or viral pathogens such as HPAI.*
		**4 ARTICLES**	
		C002, A001, A018, A046	
	FA001	Wet markets	*Live-animal markets, with their mix of wild and farmed species, sometimes involving on-site slaughter and processing, as well as the movement and exchange of animals between stalls, are prime sites for zoonotic outbreaks. The regions specified are North Africa and Southeast Asia, particularly in the case of viral pathogens such as COVID-19 or HPAI.*
		**3 ARTICLES**	
		A001, C001, A005	
	FA032	Industrialization of the agri-food sector	*Since the end of the last century, and despite advances in food hygiene, the number of deaths linked to food-borne zoonoses has remained stable after a sharp increase at the start of the industrial era. The whole world, without any particular distinction, is concerned.*
		**2 ARTICLES**	
		A019, C048	

Category	Code	Factor	Description	Articles	References
ECONOMIC	FA005	Slaughter or discount sale of sick animals or their meat	*This practice consists of evacuating as quickly as possible any animal suspected of being ill, either to another farm or to the consumer market. The main region specified is South-East Asia, particularly in the case of bacterial pathogens such as Bacillus anthracis.*	**2 ARTICLES**	A001, C005
ECONOMIC	FA011	Cost of specific therapies too high for hospital systems in developing or underdeveloped countries (here, postexposure prophylaxis for rabies)	*The cost of Post-Exposure Prophylaxis (PEP) for Rabies, for example, may represent too great a burden for hospital systems that do not have the financial means to deploy the service on a large scale. The region specified is South-East Asia.*	**1 ARTICLE**	A004
ECONOMIC	FA048	Differential funding for zoonosis management depending on the disease (with a particular preference for malaria)	*Certain well-known diseases lead to a preferential capture of funds allocated to Public Health. For example, in countries where malaria is endemic, funds earmarked for the fight against arboviroses can be siphoned off by malaria projects, which is a risk factor for neglected diseases. The main region specified is South-East Asia.*	**1 ARTICLE**	A064
ECONOMIC	FA014	Illegal imports of meat or uncontrolled meat	*Illegal imports of uncontrolled meat (in this case, horsemeat, pork, wild boar and bear meat) present a risk of introducing zoonoses, particularly food-borne ones. The main region specified is Europe, particularly in the case of endoparasitic pathogens such as Trichinella spiralis.*	**1 ARTICLE**	A008
SOCIAL	FA018	Lack of public knowledge about zoonotic risk factors due to a lack of communication and awareness-raising or misinformation	*Lack of media coverage, misinformation or disinformation on social networks, and lack of education and awareness-raising, all contribute to the spread of epidemics. Areas specified can be found all over the world, but particularly in South and East Africa, Central and South America and the Caribbean.*	**21 ARTICLES**	A009, C010, C011, C012, C013, C014, C015, C017, C018, A011, A014, C023, C024, C025, C026, C027, C028, C029, C030, C031, C032
SOCIAL	FA022	Belonging to a neglected or stigmatized minority	*Lack of education and poverty, due to belonging to a minority or a community that is neglected or even stigmatized and rejected, is a barrier to prevention. Marginalized or indigenous populations with a lack of access to health services are populations at high health risk. Areas specified are found throughout the world, but particularly in Central and South America and the Caribbean.*	**17 ARTICLES**	A014, C024, C030, C033, C034, C035, C036, C037, A019, C052, A028, C122, C123, C124, C125, C126, C127

Category	Code	Factor	Description	Number of articles	Articles
SOCIAL	FA010	Poor governance due to lack of political will, lack of resources, degraded or inadequate public health measures	*When public authorities allocate little or no budget, implement no prevention, prophylaxis or control measures, the risk is increased. The resilience of public health systems influences the emergence and evolution of epidemics. The same applies to poorly prepared surveillance, governance and action plans in public health systems. Poor resource allocation, a lack of large-scale prevention programs and a lack of knowledge among policy-makers reduce the effectiveness of the response. Areas specified can be found all over the world, but South America and South-East Asia more particularly, especially in the case of viral pathogens such as rabies.*	**15 ARTICLES**	A004, A009, C009, C010, C011, C012, C013, C014, C015, C016, C017, C018, A014, C030, C040
SOCIAL	FA016	War, famine, political instability and conflict	*Periods of war and famine encourage infections, due to their economic and environmental impact. They also create favorable social conditions for the development of zoonoses. Zones specified are found throughout the world, but particularly in South Asia and the Indian Ocean, notably in the case of bacterial pathogens such as Francisella tularensis and Brucella spp. or viral pathogens such as CCHFV.*	**14 ARTICLES**	A009, C010, C011, C012, C013, C014, C015, C017, C018, A019, C049, C050, C051, A025
SOCIAL	FA003	Consumption of contaminated or high-risk food products in daily life	*Certain dietary habits, whether cultural or linked to food insecurity, present an increased risk. These include raw date palm sap (when contaminated by fruit bats), raw milk products, fetuses and placentas, barbecued meat or undercooked pork, raw seafood, bushmeat, especially hippopotamus, and fermented products made from raw fish. Drinking untreated water from surface sources such as lakes, ponds, rivers, etc... when hiking or camping, for example, presents a risk. Specifed areas can be found all over the world, but particularly in South and East Africa, North America, Central and South America and the Caribbean, South-East Asia, South Asia and the Indian Ocean, or Europe, particularly in the case of bacterial pathogens such as Brucella spp., Campylobacter spp. and Bacillus anthracis, or viral pathogens such as Nipah.*	**12 ARTICLES**	A001, C003, C004, A014, A018, A019, C045, C046, C047, A025, A029, A062
SOCIAL	FA017	Declining public confidence in government public health interventions, especially vaccination	*Reduced confidence in the government and its actions amplifies reaction and reactionary narratives, favoring the appearance and spread of epidemics. Confidence in vaccination and its accessibility influence the appearance and evolution of epidemics. The whole world, without any particular distinction, is concerned.*	**10 ARTICLES**	A009, C009, C010, C011, C012, C013, C014, C015, C017, C018

Category	Code	Factor	Description
SOCIAL	FA029	Ecotourism and international travel activities	*Ecotourism activities (camping in the forest, contact with wild animals at the zoo, rafting, caving in bat caves...) or, more directly, international travel and tourist flows, increase the risk. Specifed areas are found all over the world, but particularly in Africa, Central and South America and the Caribbean.*
		**7 ARTICLES**	A019, C041, C042, C043, C046, A032, A050
	FA023	Lack of knowledge and training among health care professionals	*A low level of risk perception or overconfidence on the part of professionals represents a risk. For example: the great homogeneity of arbovirus symptoms and the lack of diagnostic resources lead to misdiagnosis of the etiology of diseases, and are risk factors in the emergence of a zoonotic outbreak in the event of poor knowledge of the diseases. Specifed areas are found throughout the world, but particularly in South America, especially in the case of arboviral pathogens.*
		**6 ARTICLES**	A014, C027, C029, C033, C038, A064
	FA041	Colonial practices and illegal activities in indigenous territories	*Colonial practices (destabilization of communities) often combined with illegal activities in indigenous territories (mining, logging, poaching) are risk factors specifically for these populations. The whole world, without any particular distinction, is concerned.*
		**4 ARTICLES**	A028, C078, C079, C080
	FA024	Disparities in knowledge and communication problems between professionals	*Language and communication barriers, as well as differences in knowledge between the various scientific, administrative and other players, reduce the effectiveness of prevention interventions. The main region specified is Central and South America and the Caribbean.*
		**4 ARTICLES**	A014, C024, C029, C039
	FA031	Pet ownership	*The ownership of pets, i.e. animals bred to desire close contact with humans, or the ownership of New Pets (NACs: here, especially among young people), and especially when animals are kept indoors overnight, is a risk factor. Specifed areas are found all over the world, but particularly in South and East Africa, especially in the case of bacterial pathogens such as Salmonella spp. or endoparasitic agents such as Enterocytozoon bieneusi.*
		**4 ARTICLES**	A019, C047, A021, A038

Category	Code	Factor	Description	Articles
SOCIAL	FA006	Poor butchery practices and management of butchery waste and carcasses	*Poor butchering and meat management practices, as well as poor management of carcasses (dumping in fields, rivers and canals), lead to contamination of people, pastures and the environment. The regions specified are Central and West Africa and South-East Asia, particularly in the case of bacterial pathogens such as Bacillus anthracis, or viral agents such as COVID-19.*	**3 ARTICLES**: A001, C006, A005
SOCIAL	FA027	Religious and traditional beliefs involving contact with animals or the consumption of contaminated or risky food products within a ritual or traditional event	*Consumption of high-risk food products as part of occasional ritual and/or festive practices is a risk factor. For example, the consumption of raw pork as part as of a festive ritual, or as part of a man's sense of virility. In some cases, however, religious prohibitions can act as a protective factor. The regions specified are Central and West Africa and South-East Asia, particulary in the case of endoparasitic pathogens such as Taenia solium.*	**ARTICLES**: A015, A046, A053
SOCIAL	FA008	Societal-level gender bias in the access, production and dissemination of information regarding risky practices and the distribution of risky tasks	*The gendered distribution of tasks within pastoral communities leads to an asymmetry in the performance of tasks but also in the production, dissemination, and access to information about risks and risky practices, thus accentuating the risk in certain parts of population. Men are more often assigned to tasks presenting a zoonotic risk and women have less access to information. Regions specified are Central and West Africa, South and East Africa, particularly in the case of bacterial pathogens such as Brucella abortis.*	**2 ARTICLES**: A002, A055
SOCIAL	FA026	Livestock in free circulation in the residential area	*Animals, particularly pigs, moving freely in residential areas, and thus with free access to household and human waste, increase the probability for a parasite to complete its cycle. Regions specified are South and East Africa and South-East Asia, particularly in the case of endoparasitic pathogens such as Taenia solium.*	**2 ARTICLES**: A015, A017
SOCIAL	FA047	Negative cultural influence on care-seeking	*A person's culture can influence the way in which they seek care and contact health infrastructures. Some cultures refuse medication until the signs are very marked, and lead to reluctance to take medication. The main region specified is Southeast Asia.*	**1 ARTICLE**: A053

SOCIAL	FA046	Political and socioeconomic organizations of society that limit the implementation of restrictive, individual, preventive measures	*The socio-economic organization of societies may disadvantage the implementation of hand washing or social distancing. "Democratic" and "open" countries, with fewer personal restrictions, are more at risk. The regions specified cover all of Africa.*
		**1 ARTICLE**	
		A050	

Table A.3 - Articles included and their links. Articles included through the search phase are annotated in green with an identifier starting by A. Complementary articles are annotated in pink with an identifier starting by C and are linked to their source by its identifier.

CODE	AUTHORS	TITLE	DOI	JOURNAL	LINK
A001	Chowdhury S, Aleem MA, Khan MSI, Hossain ME, Ghosh S, Rahman MZ	*Major zoonotic diseases of public health importance in Bangladesh*	10.1002/vms3.465	*Veterinary Medicine and Science*	
A002	Coyle AH, Berrian AM, Van Rooyen J, Bagnol B, Smith MH	*Gender Roles and One Health Risk Factors at the Human-Livestock-Wildlife Interface, Mpumalanga Province, South Africa*	10.1007/s10393-020-01478-9	*EcoHealth*	
A003	Kanouté YB, Gragnon BG, Schindler C, Bonfoh B, Schelling E.	*Reprint of "Epidemiology of brucellosis, Q Fever and Rift Valley Fever at the human and livestock interface in northern Cote d Ivoire"*	10.1016/j.actatropica.2017.08.013	*Acta Tropica*	
A004	Ahmad W, Naeem MA, Akram Q, Ahmad S, Younus M.	*Exploring rabies endemicity in Pakistan: Major constraints & possible solutions*	10.1016/j.actatropica.2021.106011	*Acta Tropica*	
A005	Alhaji NB, Odetokun IA, Lawan MK, Adeiza AM, Nafarnda WD, Salihu MJ.	*Risk assessment and preventive health behaviours toward COVID-19 amongst bushmeat handlers in Nigerian wildlife markets: Drivers and One Health challenge*	10.1016/j.actatropica.2022.106621	*Acta Tropica*	
A006	De Oca-Aguilar ACM, Rebollar-Téllez EA, Sosa-Bibiano EI, López-Avila KB, Torres-Castro JR, Loría-Cervera EN	*Effect of land use change on the phlebotomine sand fly assemblages in an emergent focus of cutaneous leishmaniasis in Yucatan, Mexico*	10.1016/j.actatropica.2022.106628	*Acta Tropica*	
A007	Harrison ME, Cheyne SM, Darma F, Ribowo DA, Limin SH, Struebig MJ	*Hunting of flying foxes and perception of disease risk in Indonesian Borneo*	10.1016/j.biocon.2011.06.021	*Biological Conservation*	
A008	Pozio E	*The impact of globalization and climate change on Trichinella spp. Epidemiology*	10.1016/j.fawpar.2022.e00154	*Food and Waterborne Parasitology*	
A009	Kousoulis AA, Grant I	*SPEECH: A literature based framework for the study of past epidemics*	10.1016/j.jiph.2022.01.010	*Journal of Infection and Public Health*	

A010	Min K-D, Schneider MC, Cho S	*Association between predator species richness and human hantavirus infection emergence in Brazil*	10.1016/j.onehlt.2020.100196	*One Health*	
A011	Alemayehu G, Mamo G, Desta H, Alemu B, Wieland B	*Knowledge, attitude, and practices to zoonotic disease risks from livestock birth products among smallholder communities in Ethiopia*	10.1016/j.onehlt.2021.100223	*One Health*	
A012	Zamir L, Baum M, Bardenstein S, et al.	*The association between natural drinking water sources and the emergence of zoonotic leptospirosis among grazing beef cattle herds during a human outbreak*	10.1016/j.onehlt.2022.100372	*One Health*	
A013	De Moura RR, Chiba De Castro WA, Farinhas JH, et al.	*One Health Index (OHI) applied to Curitiba, the ninth-largest metropolitan area of Brazil, with concomitant assessment of animal, environmental, and human health indicators*	10.1016/j.onehlt.2022.100373	*One Health*	
A014	Palomares Velosa JE, Riaño Sánchez S, Martínez Marín A, Cediel Becerra NM	*Prevention of exposure to zoonoses in rural Latin America: Social ecological factors in a diverse regional context*	10.1016/j.onehlt.2022.100444	*One Health*	
A015	Bardosh K, Inthavong P, Xayaheuang S, Okello AL.	*Controlling parasites, understanding practices: the biosocial complexity of a One Health intervention for neglected zoonotic helminths in northern Lao PDR*	10.1016/j.socscimed.2014.09.030	*Social Science & Medicine*	
A016	Saylors KE, Mouiche MM, Lucas A, et al.	*Market characteristics and zoonotic disease risk perception in Cameroon bushmeat markets*	10.1016/j.socscimed.2020.113358	*Social Science & Medicine*	
A017	Thys S, Mwape KE, Lefèvre P, et al.	*Why pigs are free-roaming: Communities perceptions, knowledge and practices regarding pig management and taeniosis/cysticercosis in a Taenia solium endemic rural area in Eastern Zambia*	10.1016/j.vetpar.2016.05.029	*Veterinary Parasitology*	

A018	Kapperud G, Espeland G, Wahl E, et al.	*Factors associated with increased and decreased risk of Campylobacter infection: A prospective case-control study in Norway*	10.1093/aje/kwg139	*American Journal of Epidemiology*	
A019	Cascio A, Bosilkovski M, Rodriguez-Morales AJ, Pappas G	*The socio-ecology of zoonotic infections*	10.1111/j.1469-0691.2010.03451.x	*Clinical Microbiology and Infection*	
A020	Williams PC, Bartlett AW, Howard-Jones A, et al.	*Impact of climate change and biodiversity collapse on the global emergence and spread of infectious diseases*	10.1111/jpc.15681	*Journal of Paediatrics and Child Health*	
A021	Barnes AN, Mumma J, Cumming O	*Role, ownership and presence of domestic animals in peri-urban households of Kisumu, Kenya*	10.1111/zph.12429	*Zoonoses and Public Health*	
A022	McMahon BJ, Morand S, Gray JS	*Ecosystem change and zoonoses in the Anthropocene*	10.1111/zph.12489	*Zoonoses and Public Health*	
A023	Winck GR, Raimundo RLG, Fernandes-Ferreira H, et al.	*Socioecological vulnerability and the risk of zoonotic disease emergence in Brazil*	10.1126/sciadv.abo5774	*Science Advances*	
A024	Patz JA, Daszak P, Tabor GM, et al.	*Unhealthy landscapes: Policy recommendations on land use change and infectious disease emergence*	10.1289/ehp.6877	*Environmental Health Perspectives*	
A025	Bagheri Nejad R, Krecek RC, Khalaf OH, Hailat N, Arenas-Gamboa AM.	*Brucellosis in the Middle East: Current situation and a pathway forward*	10.1371/journal.pntd.0008071	*PLoS Neglected Tropical Diseases*	
A026	Morgan J, Strode C, Salcedo-Sora JE.	*Climatic and socio-economic factors supporting the co-circulation of dengue, Zika and chikungunya in three different ecosystems in Colombia*	10.1371/journal.pntd.0009259	*PLoS Neglected Tropical Diseases*	
A027	Barnes AN, Davaasuren A, Baasandavga U, Lantos PM, Gonchigoo B, Gray GC	*Zoonotic enteric parasites in mongolian people, animals, and the environment: Using one health to address shared pathogens*	10.1371/journal.pntd.0009543	*PLoS Neglected Tropical Diseases*	
A028	Ellwanger JH, Fearnside PM, Ziliotto M, et al.	*Synthesizing the connections between environmental disturbances and zoonotic spillover*	10.1590/0001-3765202220211530	*Anais da Academia Brasileira de Ciencias*	

**A029**	Lehman MW, Craig AS, Malama C, et al.	*Role of Food Insecurity in Outbreak of Anthrax Infections among Humans and Hippopotamuses Living in a Game Reserve Area, Rural Zambia*	10.3201/eid2309.161597	*Emerging Infectious Diseases*	
**A030**	Rush ER, Dale E, Aguirre AA	*Illegal Wildlife Trade and Emerging Infectious Diseases: Pervasive Impacts to Species, Ecosystems and Human Health*	10.3390/ani11061821	*Animals*	
**A031**	Kyung-Duk M, Ju-Yeun L, Yeonghwa S, Sung-il C	*Deforestation increases the risk of scrub typhus in Korea*	10.3390/ijerph16091518	*International Journal of Environmental Research and Public Health*	
**A032**	Muehlenbein MP, Dore KM, Gassen J, Nguyen V, Jolley OG, Gallagher C	*Travel medicine meets conservation medicine in St. Kitts: Disinhibition, cognitive-affective inconsistency, and disease risk among vacationers around green monkeys (Chlorocebus sabaeus)*	10.1002/ajp.23301	*American Journal of Primatology*	
**A033**	Dudley JP, Hoberg EP, Jenkins EJ, Parkinson AJ	*Climate Change in the North American Arctic: A One Health Perspective*	10.1007/s10393-015-1036-1	*EcoHealth*	
**A034**	Rengifo-Correa L, Rocha-Ortega M, Córdoba-Aguila	*Modeling Mosquitoes and their Potential Odonate Predators Under Different Land Uses*	10.1007/s10393-022-01600-z	*EcoHealth*	
**A035**	Singh BB, Gajadhar AA	*Role of India s wildlife in the emergence and re-emergence of zoonotic pathogens, risk factors and public health implications*	10.1016/j.actatropica.2014.06.009	*Acta Tropica*	
**A036**	Everard M, Johnston P, Santillo D, Staddon C	*The role of ecosystems in mitigation and management of Covid-19 and other zoonoses*	10.1016/j.envsci.2020.05.017	*Environmental Science & Policy*	
**A037**	Asokan GV, Vanitha A	*Disaster response under One Health in the aftermath of Nepal earthquake, 2015*	10.1016/j.jegh.2016.03.001	*Journal of Epidemiology and Global Health*	

A038	Li W, Xiao L	*Ecological and public health significance of Enterocytozoon bieneusi*	10.1016/j.onehlt.2020.100209	*One Health*	
A039	Walsh MG, Bhat R, Nagarajan-Radha V, et al.	*Low mammalian species richness is associated with Kyasanur Forest disease outbreak risk in deforested landscapes in the Western Ghats, India*	10.1016/j.onehlt.2021.100299	*One Health*	
A040	Jagadesh S, Combe M, Ginouvès M, et al.	*Spatial variations in Leishmaniasis: A biogeographic approach to mapping the distribution of Leishmania species*	10.1016/j.onehlt.2021.100307	*One Health*	
A041	De Almeida Soares D, Arcêncio RA, Fronteira I	*Inequities between migrants and non-migrants with TB: Surveillance evidence from the Brazilian border State of Roraima*	10.1016/j.onehlt.2022.100473	*One Health*	
A042	Chimera ET, Fosgate GT, Etter EMC, Boulangé A, Vorster I, Neves L	*A one health investigation of pathogenic trypanosomes of cattle in Malawi*	10.1016/j.prevetmed.2020.105255	*Preventive Veterinary Medicine*	
A043	Fornace KM, Brock PM, Abidin TR, et al.	*Environmental risk factors and exposure to the zoonotic malaria parasite Plasmodium knowlesi across northern Sabah, Malaysia: a population-based cross-sectional survey*	10.1016/S2542-5196(19)30045-2	*The Lancet Planetary Health*	
A045	Tounta DD, Nastos PT, Tesseromatis C	*Human activities and zoonotic epidemics: a two-way relationship. The case of the COVID-19 pandemic*	10.1017/sus.2022.18	*Global Sustainability*	
A046	Lawson ET, Ohemeng F, Ayivor J, Leach M, Waldman L, Ntiamoa-Baidu Y	*Understanding framings and perceptions of spillover Preventing future outbreaks of bat-borne zoonoses*	10.1108/DPM-04-2016-0082	*Disaster Prevention and Management: An International Journal*	
A047	Brumfield KD, Usmani M, Chen KM, et al.	*Environmental parameters associated with incidence and transmission of pathogenic Vibrio spp.*	10.1111/1462-2920.15716	*Environmental Microbiology*	

**A048**	Murray DL, Kapke CA, Evermann JF, Fuller TK	*Infectious disease and the conservation of free-ranging large carnivores*	10.1111/j.1469-1795.1999.tb00070.x	*Animal Conservation*	
**A049**	LaDeau SL	*Rodents harbouring zoonotic pathogens take advantage of abandoned land in post-Katrina New Orleans*	10.1111/mec.15843	*Molecular Ecology*	
**A050**	Gesesew HA, Koye DN, Fetene DM, et al.	*Risk factors for COVID-19 infection, disease severity and related deaths in Africa: a systematic review*	10.1136/bmjopen-2020-044618	*BMJ Open*	
**A051**	Rocque RJ, Beaudoin C, Ndjaboue R, et al.	*Health effects of climate change: an overview of systematic reviews*	10.1136/bmjopen-2020-046333	*BMJ Open*	
**A052**	Myers SS, Patz JA	*Emerging Threats to Human Health from Global Environmental Change*	10.1146/annurev.environ.033108.102650	*Annual Review of Environment and Resources*	
**A053**	Burniston S, Okello AL, Khamlome B, et al.	*Cultural drivers and health-seeking behaviours that impact on the transmission of pig-associated zoonoses in Lao People s Democratic Republic*	10.1186/2049-9957-4-11	*Infectious Diseases of Poverty*	
**A054**	Kabululu ML, Ngowi HA, Mlangwa JED, et al.	*Endemicity of Taenia solium cysticercosis in pigs from Mbeya Rural and Mbozi districts, Tanzania*	10.1186/s12917-020-02543-9	*BMC Veterinary Research*	
**A055**	Babo SAY, Fokou G, Yapi RB, et al.	*Gendered asymmetry of access to knowledge for brucellosis control among pastoral communities in north-west Cote d Ivoire*	10.1186/s13570-022-00241-9	*Pastoralism*	
**A056**	Zhu G-L, Tang Y-Y, Limpanont Y, Wu Z-D, Li J, Lv Z-Y.	*Zoonotic parasites carried by invasive alien species in China*	10.1186/s40249-018-0512-6	*Infectious Diseases of Poverty*	
**A057**	Aguirre AA, Tabor GM	*Global Factors Driving Emerging Infectious Diseases Impact on Wildlife Populations*	10.1196/annals.1428.052	*Annals of the New York Academy of Sciences*	

A058	Combe M, Gozlan RE, Jagadesh S, et al.	*Comparison of Mycobacterium ulcerans (Buruli ulcer) and Leptospira sp. (Leptospirosis) dynamics in urban and rural settings*	10.1371/journal.pntd.0007074	*PLoS Neglected Tropical Diseases*	
A059	Barnes AN, Davaasuren A, Baasandagva U, Gray GC	*A systematic review of zoonotic enteric parasitic diseases among nomadic and pastoral people*	10.1371/journal.pone.0188809	*PLoS ONE*	
A060	Lima VFS, Ramos RAN, Giannelli A, et al.	*Zoonotic parasites in wild animals such as carnivores and primates that are traded illegally in Brazil*	10.29374/2527-2179.bjvm113720	*Brazilian Journal of Veterinary Medicine*	
A061	Aguirre AA, Gore ML, Kammer-Kerwick M, et al.	*Opportunities for Transdisciplinary Science to Mitigate Biosecurity Risks From the Intersectionality of Illegal Wildlife Trade With Emerging Zoonotic Pathogens*	10.3389/fevo.2021.604929	*Frontiers in Ecology and Evolution*	
A062	Keatts LO, Robards M, Olson SH, et al.	*Implications of Zoonoses From Hunting and Use of Wildlife in North American Arctic and Boreal Biomes: Pandemic Potential, Monitoring, and Mitigation*	10.3389/fpubh.2021.627654	*Frontiers in Public Health*	
A063	Douglas KO, Payne K, Sabino-Santos G, Agard J.	*Influence of Climatic Factors on Human Hantavirus Infections in Latin America and the Caribbean: A Systematic Review*	10.3390/pathogens11010015	*Pathogens*	
A064	Tajudeen YA, Oladunjoye IO, Mustapha MO, Mustapha ST, Ajide-Bamigboye NT	*Tackling the global health threat of arboviruses: An appraisal of the three holistic approaches to health*	10.34172/hpp.2021.48	*Health Promotion Perspectives*	
A065	Lancelot R, Béral M, Rakotoharinome VM, et al.	*Drivers of Rift Valley fever epidemics in Madagascar*	10.1073/pnas.1607948114	*Proceedings of the National Academy of Sciences*	
A066	Pinter A, Prist PR, Marrelli MT	*Biodiversity and public health interface*	10.1590/1676-0611-BN-2021-1280	*Biota Neotropica*	

C001	Khan SU, Gurley ES, Gerloff N, et al.	*Avian influenza surveillance in domestic waterfowl and environment of live bird markets in Bangladesh, 2007–2012*	10.1038/s41598-018-27515-w	*Scientific Reports*	A001
C002	Nasreen S, Khan SU, Luby SP, et al.	*Highly Pathogenic Avian Influenza A(H5N1) Virus Infection among Workers at Live Bird Markets, Bangladesh, 2009–2010*	10.3201/eid2104.141281	*Emerging Infectious Diseases*	A001
C003	Salah Uddin Khan M, Hossain J, Gurley ES, Nahar N, Sultana R, Luby SP	*Use of infrared camera to understand bats' access to date palm sap: Implications for preventing Nipah virus transmission.*	10.1007/s10393-010-0366-2	*EcoHealth*	A002
C004	Luby S, Rahman M, Hossain M, et al.	*Foodborne transmission of Nipah virus, Bangladesh.*	10.3201/eid1212.060732	*Emerging Infectious Diseases*	A003
C005	Islam MdS, Hossain MJ, Mikolon A, et al.	*Risk practices for animal and human anthrax in Bangladesh: An exploratory study*	10.3402/iee.v3i0.21356	*Infection Ecology & Epidemiology*	A004
C006	Chakraborty A	*Anthrax outbreaks in Bangladesh: An update.*	No DOI	*Health and Science Bulletin*	A005
C007	Ghosh S, Chowdhury S, Haider N, et al.	*Awareness of rabies and response to dog bites in a Bangladesh community*	10.1002/vms3.30	*Veterinary Medicine and Science*	A006
C008	Guo F, Bonebrake TC, Gibson L	*Land-Use Change Alters Host and Vector Communities and May Elevate Disease Risk*	10.1007/s10393-018-1336-3	*EcoHealth*	A006
C009	Farmer P	*Social inequalities and emerging infectious diseases.*	10.3201/eid0204.960402	*Emerging Infectious Diseases*	A009
C010	Stephens DS	*Emerging and Reemerging Infectious Diseases: A Multidisciplinary Perspective*	10.1016/S0002-9629(15)40280-0	*The American Journal of the Medical Sciences*	A009
C011	Morens DM, Folkers GK, Fauci AS	*The challenge of emerging and re-emerging infectious diseases (Erratum)*	10.1038/nature02759	*Nature*	A009
C012	Phua K-L, Lee LK	*Meeting the challenge of epidemic infectious disease outbreaks: an agenda for research.*	10.1057/palgrave.jphp.3200001	*Journal of Public Health Policy*	A009

C013	Morens DM, Folkers GK, Fauci AS	*Emerging infections: a perpetual challenge*	10.1016/S1473-3099(08)70256-1	*The Lancet Infectious Diseases*	A009
C014	Brien S, Kwong JC, Buckeridge DL	*The determinants of 2009 pandemic A/H1N1 influenza vaccination: A systematic review*	10.1016/j.vaccine.2011.12.089	*Vaccine*	A009
C015	Lowcock EC, Rosella LC, Foisy J, McGeer A, Crowcroft N	*The Social Determinants of Health and Pandemic H1N1 2009 Influenza Severity*	10.2105/AJPH.2012.300814	*American Journal of Public Health*	A009
C016	Antonis A. Kousoulis, Theodoros N. Sergentanis, Sotirios Tsiodras	*2009 H1N1 flu pandemic among professional basketball players: data from 18 countries*	No DOI	*Le Infezioni in Medicina*	A009
C017	Quinn SC, Kumar S	*Health Inequalities and Infectious Disease Epidemics: A Challenge for Global Health Security*	10.1089/bsp.2014.0032	*Biosecurity and Bioterrorism: Biodefense Strategy, Practice, and Science*	A009
C018	Grantz KH, Rane MS, Salje H, Glass GE, Schachterle SE, Cummings DAT	*Disparities in influenza mortality and transmission related to sociodemographic factors within Chicago in the pandemic of 1918*	10.1073/pnas.1612838113	*Proceedings of the National Academy of Sciences*	A009
C019	Suzán G, Marcé E, Giermakowski JT, et al.	*The Effect of Habitat Fragmentation and Species Diversity Loss on Hantavirus Prevalence in Panama*	10.1196/annals.1428.063	*Annals of the New York Academy of Sciences*	A010
C020	Prist PR, Uriarte M, Tambosi LR, et al.	*Landscape, Environmental and Social Predictors of Hantavirus Risk in São Paulo, Brazil*	10.1371/journal.pone.0163459	*PLoS ONE*	A010
C021	Lau CL, Smythe LD, Craig SB, Weinstein P.	*Climate change, flooding, urbanisation and leptospirosis: fuelling the fire?*	10.1016/j.trstmh.2010.07.002	*Transactions of The Royal Society of Tropical Medicine and Hygiene*	A012
C022	Zhang X-X, Liu J-S, Han L-F, et al.	*Towards a global One Health index: a potential assessment tool for One Health performance*	10.1186/s40249-022-00979-9	*Infectious Diseases of Poverty*	A013

**C023**	Carnero AM, Kitayama K, Diaz DA, et al.	*Risk for interspecies transmission of zoonotic pathogens during poultry processing and pork production in Peru: A qualitative study*	10.1111/zph.12463	*Zoonoses and Public Health*	A014
**C024**	Cerón A, Ortiz MR, Álvarez D, Palmer GH, Cordón-Rosales C	*Local disease concepts relevant to the design of a community-based surveillance program for influenza in rural Guatemala*	10.1186/s12939-016-0359-z	*International Journal for Equity in Health*	A014
**C025**	McGwin G, Aung M, Ervin G, Keenan J, Jolly P	*Risk Factors for Clinical Leptospirosis from Western Jamaica*	10.4269/ajtmh.2010.09-0609	*The American Journal of Tropical Medicine and Hygiene*	A014
**C026**	Ramos-Peña Y, Collazo G, Cabrera L, Martinez L, Oberhelman R, Bernabe-Ortiz A	*Free-Ranging Chickens in Households in a Periurban Shantytown in Peru—Attitudes and Practices 10 Years after a Community-Based Intervention Project*	10.4269/ajtmh.12-0760	*The American Journal of Tropical Medicine and Hygiene*	A014
**C027**	Ricardo T, Bergero LC, Bulgarella EP, Previtali MA	*Knowledge, attitudes and practices (KAP) regarding leptospirosis among residents of riverside settlements of Santa Fe, Argentina*	10.1371/journal.pntd.0006470	*PLoS Neglected Tropical Diseases*	A014
**C028**	Shapiro HG, Wilcox AS, Tate M, Willcox EV	*Can Farmers and Bats Co-exist? Farmer Attitudes, Knowledge, and Experiences with Bats in Belize*	10.26077/5wwp-sp53	*The American Journal of Tropical Medicine and Hygiene*	A014
**C029**	Rivière-Cinnamond A, Santandreu A, Luján A, et al.	*Identifying the social and environmental determinants of plague endemicity in Peru: insights from a case study in Ascope, La Libertad*	10.1186/s12889-018-5062-0	*BMC Public Health*	A014
**C030**	Glasgow L, Worme A, Keku E, Forde M	*Knowledge, attitudes, and practices regarding rabies in Grenada*	10.1371/journal.pntd.0007079	*PLoS Neglected Tropical Diseases*	A014
**C031**	Gonçalves DD, Benitez A, Lopes-Mori FMR, et al.	*Zoonoses in humans from small rural properties in Jataizinho, Parana, Brazil*	10.1590/S1517-838220130050000011	*Brazilian Journal of Microbiology*	A014

C032	Heffernan C, Thomson K, Nielsen L	*Livestock vaccine adoption among poor farmers in Bolivia: Remembering innovation diffusion theory*	10.1016/j.vaccine.2008.02.045	*Vaccine*	A014
C033	Allwood P, Muñoz-Zanzi C, Chang M, Brown PD	*Knowledge, perceptions, and environmental risk factors among Jamaican households with a history of leptospirosis*	10.1016/j.jiph.2014.03.004	*Journal of Infection and Public Health*	A014
C034	Mareze M, Benitez ADN, Brandão APD, et al.	*Socioeconomic vulnerability associated to Toxoplasma gondii exposure in southern Brazil*	10.1371/journal.pone.0212375	*PLoS ONE*	A014
C035	Arias Caicedo MR, Xavier DDA, Arias Caicedo CA, Andrade E, Abel I	*Epidemiological scenarios for human rabies exposure notified in Colombia during ten years: A challenge to implement surveillance actions with a differential approach on vulnerable populations*	10.1371/journal.pone.0213120	*PLoS ONE*	A014
C036	Peña-Quistial MG, Benavides-Montaño JA, Duque NJR, Benavides-Montaño GA	*Prevalence and associated risk factors of Intestinal parasites in rural high-mountain communities of the Valle del Cauca—Colombia*	10.1371/journal.pntd.0008734	*PLoS Neglected Tropical Diseases*	A014
C037	McCune S, Arriola CS, Gilman RH, et al	*Interspecies interactions and potential Influenza A virus risk in small swine farms in Peru*	10.1186/1471-2334-12-58	*BMC Infectious Diseases*	A014
C038	Roess A, Leibler JH, Graham JP, Lowenstein C, Waters WF	*Animal Husbandry Practices and Perceptions of Zoonotic Infectious Disease Risks Among Livestock Keepers in a Rural Parish of Quito, Ecuador*	10.4269/ajtmh.16-0485	*The American Journal of Tropical Medicine and Hygiene*	A014
C039	Costa LJCD, Fernandes MEB	*Rabies: Knowledge and Practices Regarding Rabies in Rural Communities of the Brazilian Amazon Basin*	10.1371/journal.pntd.0004474	*PLoS Neglected Tropical Diseases*	A014
C040	Dantas-Torres F, Oliveira-Filho EFD	*Human exposure to potential rabies virus transmitters in Olinda, State of Pernambuco, between 2002 and 2006*	10.1590/S0037-86822007000600003	*Revista da Sociedade Brasileira de Medicina Tropical*	A014
C041	Jensenius M, Fournier PE, Raoult	*Rickettsioses and the International Traveler*	10.1086/425365	*Clinical Infectious Diseases*	A019

C042	Pappas G, Papadimitriou P, Siozopoulou V, Christou L, Akritidis N	*The globalization of leptospirosis: worldwide incidence trends*	10.1016/j.ijid.2007.09.011	*International Journal of Infectious Diseases*	A019
C043	Beeching NJ, Fletcher TE, Hill DR, Thomson GL	*Travellers and viral haemorrhagic fevers: what are the risks?*	10.1016/j.ijantimicag.2010.06.017	*International Journal of Antimicrobial Agents*	A019
C044	Meng XJ, Lindsay DS, Sriranganathan N	*Wild boars as sources for infectious diseases in livestock and humans*	10.1098/rstb.2009.0086	*Philosophical Transactions of the Royal Society B: Biological Sciences*	A019
C045	Butt AA, Aldridge KE, Sanders CV	*Infections related to the ingestion of seafood. Part II: parasitic infections and food safety*	10.1016/S1473-3099(04)01005-9	*The Lancet Infectious Diseases*	A019
C046	Chomel BB, Belotto A, Meslin F-X	*Wildlife, exotic pets, and emerging zoonoses*	10.3201/eid1301.060480	*Emerging Infectious Diseases*	A019
C047	Mermin J, Hutwagner L, Vugia D, et al.	*Reptiles, amphibians, and human Salmonella infection: a population-based, case–control study.*	10.1086/381594	*Clinical Infectious Diseases*	A019
C048	Blancou J, Chomel BB, Belotto A, Meslin FX	*Emerging or re-emerging bacterial zoonoses: factors of emergence, surveillance and control*	10.1051/vetres:2005008	*Veterinary Research*	A019
C049	Reintjes R	*Tularemia outbreak investigation in Kosovo: case control and environmental studies.*	10.3201/eid0801.010131	*Emerging Infectious Diseases*	A019
C050	Maltezou HC, Papa A	*Crimean-Congo hemorrhagic fever: risk for emergence of new endemic foci in Europe?*	10.1016/j.tmaid.2010.04.008	*Travel Medicine and Infectious Disease*	A019
C051	Pappas G, Papadimitriou P, Akritidis N, Christou L, Tsianos EV	*The new global map of human brucellosis*	10.1016/S1473-3099(06)70382-6	*The Lancet Infectious Diseases*	A019
C052	Hotez PJ	*Neglected Infections of Poverty in the United States of America*	10.1371/journal.pntd.0000256	*PLoS Neglected Tropical Diseases*	A019
C053	Ramasamy R	*Zoonotic malaria – Global overview and research and policy needs.*	10.3389/fpubh.2014.00123	*Frontiers of Public Health*	A022

C054	Epstein JH, Field HE, Luby S, Pulliam JRC, Daszak P	*Nipah virus: Impact, origins, and causes of emergence.*	10.1007/s11908-006-0036-2	*Current Infectious Disease Reports*	A022
C055	Kilpatrick AM	*Globalization, Land Use, and the Invasion of West Nile Virus*	10.1126/science.1201010	*Science*	A022
C056	Matuschka FR, Spielman A	*The emergence of Lyme disease in a changing environment in North America and Central Europe*	10.1007/BF01193900	*Experimental & Applied Acarology*	A022
C057	Šumilo D, Bormane A, Asokliene L, et al.	*Socio-economic factors in the differential upsurge of tick-borne encephalitis in central and Eastern Europe*	10.1002/rmv.566	*Reviews in Medical Virology*	A022
C058	Randolph, SE, Asokliene, L, Avsic-Zupanc, T, et al.	*Variable spikes in tick-borne encephalitis incidence in 2006 independent of variable tick abundance but related to weather.*	10.1186/1756-3305-1-44	*Parasites and Vectors*	A022
C059	Field H, Young P, Yob JM, Mills J, Hall L, Mackenzie J.	*The natural history of Hendra and Nipah viruses*	10.1016/S1286-4579(01)01384-3	*Microbes and Infection*	A028
C060	Kaw Bing CHUA, Beng Hui CHUA, Chew Wen WANG.	*Anthropogenic deforestation, El Nino and the emergence of Nipah virus in Malaysia.*	No DOI	*The Malaysian journal of pathology*	A028
C061	Vaz VC, D'Andrea PS, Jansen AM.	*Effects of habitat fragmentation on wild mammal infection by Trypanosoma cruzi*	10.1017/S003118200700323X	*Parasitology*	A028
C062	Walsh MG, Mor SM, Maity H, Hossain S	*Forest loss shapes the landscape suitability of Kyasanur Forest disease in the biodiversity hotspots of the Western Ghats, India*	10.1093/ije/dyz232	*International Journal of Epidemiology*	A028
C063	Ellwanger JH, Chies JAB	*Keeping track of hidden dangers - The short history of the Sabiá virus*	10.1590/0037-8682-0330-2016	*Revista da Sociedade Brasileira de Medicina Tropical*	A028
C064	Reis S, Melo M, Covas R, et al.	*Influence of land use and host species on parasite richness, prevalence and co-infection patterns*	10.1016/j.ijpara.2020.08.005	*International Journal for Parasitology*	A028
C065	Lai-Meng LOOI, Kaw-Bing CHUA	*Lessons from the Nipah virus outbreak in Malaysia*	No DOI	*The Malaysian Journal of Pathology*	A028

C066	Rulli MC, Santini M, Hayman DTS, D'Odorico P	*The nexus between forest fragmentation in Africa and Ebola virus disease outbreaks*	10.1038/srep41613	*Scientific Reports*	A028
C067	Olivero J, Fa JE, Real R, et al.	*Recent loss of closed forests is associated with Ebola virus disease outbreaks*	10.1038/s41598-017-14727-9	*Scientific Reports*	A028
C068	Chua KB	*Nipah virus outbreak in Malaysia*	10.1016/s1386-6532(02)00268-8	*Journal of Clinical Virology*	A028
C069	Keiser J, Maltese MF, Erlanger TE, et al.	*Effect of irrigated rice agriculture on Japanese encephalitis, including challenges and opportunities for integrated vector management*	10.1016/j.actatropica.2005.04.012	*Acta Tropica*	A028
C070	Shah HA, Huxley P, Elmes J, Murray KA	*Agricultural land-uses consistently exacerbate infectious disease risks in Southeast Asia*	10.1038/s41467-019-12333-z	*Nature Communications*	A028
C071	Pradyumna A, Egal F, Utzinger J	*Sustainable food systems, health and infectious diseases: Concerns and opportunities*	10.1016/j.actatropica.2018.12.042	*Acta Tropica*	A028
C072	Da Rosa EST, Kotait I, Barbosa TFS, et al.	*Bat-transmitted Human Rabies Outbreaks, Brazilian Amazon*	10.3201/eid1208.050929	*Emerging Infectious Diseases*	A028
C073	Kuzmin IV, Bozick B, Guagliardo SA, et al.	*Bats, emerging infectious diseases, and the rabies paradigm revisited*	10.3402/ehtj.v4i0.7159	*Emerging Health Threats Journal*	A028
C074	Morand S, Lajaunie C	*Outbreaks of Vector-Borne and Zoonotic Diseases Are Associated With Changes in Forest Cover and Oil Palm Expansion at Global Scale*	10.3389/fvets.2021.661063	*Frontiers in Veterinary Science*	A028
C075	Figueiredo GGD, Borges AA, Campos GM, et al.	*Diagnosis of hantavirus infection in humans and rodents in Ribeirão Preto, State of São Paulo, Brazil*	10.1590/S0037-86822010000400002	*Revista da Sociedade Brasileira de Medicina Tropical*	A028
C076	Mills JN	*Biodiversity loss and emerging infectious disease: An example from the rodent-borne hemorrhagic fevers*	10.1080/14888386.2006.9712789	*Biodiversity*	A028

C077	Guterres A, De Oliveira RC, Fernandes J, et al.	*Co-circulation of Araraquara and Juquitiba Hantavirus in Brazilian Cerrado*	10.1007/s00248-017-1061-4	*Microbial Ecology*	A028
C078	Valeggia CR, Snodgrass JJ	*Health of Indigenous Peoples*	10.1146/annurev-anthro-102214-013831	*Annual Review of Anthropology*	A028
C079	Owers KA, Sjödin P, Schlebusch CM, Skoglund P, Soodyall H, Jakobsson M	*Adaptation to infectious disease exposure in indigenous Southern African populations*	10.1098/rspb.2017.0226	*Proceedings of the Royal Society B: Biological Sciences*	A028
C080	Brancalion PHS, Broadbent EN, de-Miguel S, et al.	*Emerging threats linking tropical deforestation and the COVID-19 pandemic.*	10.1016/j.pecon.2020.09.006	*Perspectives in Ecology and Conservation*	A028
C081	Ferrante L, Fearnside PM	*Protect Indigenous peoples from COVID-19*	10.1126/science.abc0073	*Science*	A028
C082	Ellwanger JH, Kulmann-Leal B, Kaminski VL, et al.	*Beyond diversity loss and climate change: Impacts of Amazon deforestation on infectious diseases and public health*	10.1590/0001-3765202020191375	*Anais da Academia Brasileira de Ciencias*	A028
C083	Guégan J-F, Ayouba A, Cappelle J, De Thoisy B	*Forests and emerging infectious diseases: unleashing the beast within*	10.1088/1748-9326/ab8dd7	*Environmental Research Letters*	A028
C084	Ostergard RL	*Ebola and the pestilence of corporate and governmental corruption in Guinea: Did mining interests exacerbate the largest Ebola outbreak in history (2014–2016)?*	10.1016/j.exis.2020.11.011	*The Extractive Industries and Society*	A028
C085	Rotureau B, Joubert M, Clyti E, Djossou F, Carme B	*Leishmaniasis among Gold Miners, French Guiana*	10.3201/eid1207.051466	*Emerging Infectious Diseases*	A028
C086	Terças-Trettel ACP, Oliveira ECD, Fontes CJF, et al.	*Malaria and Hantavirus Pulmonary Syndrome in Gold Mining in the Amazon Region, Brazil*	10.3390/ijerph16101852	*International Journal of Environmental Research and Public Health*	A028
C087	Eisler R	*Risks of Gold Miners: A Synoptic Review*	10.1023/A:1024573701073	*Environmental Geochemistry and Health*	A028

C088	Anyamba A, Linthicum KJ, Tucker CJ	*Climate-disease connections: Rift Valley Fever in Kenya*	10.1590/s0102-311x2001000700022	*Cadernos de Saude Publica*	A028
C089	Martin V, Chevalier V, Ceccato P, et al.	*The impact of climate change on the epidemiology and control of Rift Valley fever*	No DOI	*Revue Scientifique Et Technique (International Office of Epizootics)*	A028
C090	Mirsaeidi M, Motahari H, Taghizadeh Khamesi M, Sharifi A, Campos M, Schraufnagel DE	*Climate Change and Respiratory Infections*	10.1513/AnnalsATS.201511-729PS	*Annals of the American Thoracic Society*	A028
C091	Park BJ, Sigel K, Vaz V, et al.	*An Epidemic of Coccidioidomycosis in Arizona Associated with Climatic Changes, 1998–2001*	10.1086/430092	*The Journal of Infectious Diseases*	A028
C092	Hashizume M, Wagatsuma Y, Faruque ASG, et al.	*Factors determining vulnerability to diarrhoea during and after severe floods in Bangladesh*	10.2166/wh.2008.062	*Journal of Water and Health*	A028
C093	Wilkinson P, Smith KR, Joffe M, Haines A	*A global perspective on energy: health effects and injustices*	10.1016/S0140-6736(07)61252-5	*The Lancet*	A028
C094	Greer A, Ng V, Fisman D	*Climate change and infectious diseases in North America: the road ahead*	10.1503/cmaj.081325	*Canadian Medical Association Journal*	A028
C095	Dantas-Torres F	*Climate change, biodiversity, ticks and tick-borne diseases: The butterfly effect*	10.1016/j.ijppaw.2015.07.001	*International Journal for Parasitology: Parasites and Wildlife*	A028
C096	Wilke ABB, Beier JC, Benelli G	*Complexity of the relationship between global warming and urbanization – an obscure future for predicting increases in vector-borne infectious diseases*	10.1016/j.cois.2019.06.002	*Current Opinion in Insect Science*	A028
C097	Brubacher J, Allen DM, Déry SJ, et al.	*Associations of five food- and water-borne diseases with ecological zone, land use and aquifer type in a changing climate*	10.1016/j.scitotenv.2020.138808	*Science of The Total Environment*	A028

C098	Timofeev V, Bahtejeva I, Mironova R, et al.	*Insights from Bacillus anthracis strains isolated from permafrost in the tundra zone of Russia*	10.1371/journal.pone.0209140	*PLoS ONE*	A028
C099	Maksimovic Z, Cornwell MS, Semren O, Rifatbegovic M	*The apparent role of climate change in a recent anthrax outbreak in cattle:*	10.20506/rst.36.3.2727	*Revue Scientifique et Technique de l OIE*	A028
C100	Stella E, Mari L, Gabrieli J, Barbante C, Bertuzzo E	*Permafrost dynamics and the risk of anthrax transmission: a modelling study*	10.1038/s41598-020-72440-6	*Scientific Reports*	A028
C101	Kalinda C, Chimbari M, Mukaratirwa S	*Implications of Changing Temperatures on the Growth, Fecundity and Survival of Intermediate Host Snails of Schistosomiasis: A Systematic Review*	10.3390/ijerph14010080	*International Journal of Environmental Research and Public Health*	A028
C102	Tu C, Crameri G, Kong X, et al.	*Antibodies to SARS Coronavirus in Civets*	10.3201/eid1012.040520	*Emerging Infectious Diseases*	A028
C103	Kan B, Wang M, Jing H, et al.	*Molecular Evolution Analysis and Geographic Investigation of Severe Acute Respiratory Syndrome Coronavirus-Like Virus in Palm Civets at an Animal Market and on Farms*	10.1128/JVI.79.18.11892-11900.2005	*Journal of Virology*	A028
C104	Wang L-F, Shi Z, Zhang S, Field H, Daszak P, Eaton B	*Review of Bats and SARS*	10.3201/eid1212.060401	*Emerging Infectious Diseases*	A028
C105	Judson SD, Fischer R, Judson A, Munster VJ.	*Ecological Contexts of Index Cases and Spillover Events of Different Ebolaviruses*	10.1371/journal.ppat.1005780	*PLoS Pathogens*	A028
C106	Van Vliet N, Moreno J, Gomez J, et al.	*Bushmeat and human health: Assessing the Evidence in tropical and sub-tropical forests*	10.15451/ec2017-04-6.3-1-44	*Ethnobiology and Conservation*	A028
C107	Da Silva MB, Portela JM, Li W, et al.	*Evidence of zoonotic leprosy in Pará, Brazilian Amazon, and risks associated with human contact or consumption of armadillos*	10.1371/journal.pntd.0006532	*PLoS Neglected Tropical Diseases*	A028
C108	Walsh MG, Mor SM, Hossain S	*The elephant–livestock interface modulates anthrax suitability in India*	10.1098/rspb.2019.0179	*Proceedings of the Royal Society B: Biological Sciences*	A028

C109	Johnson CK, Hitchens PL, Pandit PS, et al.	*Global shifts in mammalian population trends reveal key predictors of virus spillover risk*	10.1098/rspb.2019.2736	*Proceedings of the Royal Society B: Biological Sciences*	A028
C110	Zhang Y-Z, Holmes EC	*A Genomic Perspective on the Origin and Emergence of SARS-CoV-2*	10.1016/j.cell.2020.03.035	*Cell*	A028
C111	Holmes EC, Goldstein SA, Rasmussen AL, et al.	*The origins of SARS-CoV-2: A critical review*	10.1016/j.cell.2021.08.017	*Cell*	A028
C112	Ibrahim M, Schelling E, Zinsstag J, Hattendorf J, Andargie E, Tschopp R.	*Sero-prevalence of brucellosis, Q-fever and Rift Valley fever in humans and livestock in Somali Region, Ethiopia*	10.1371/journal.pntd.0008100	*PLoS Neglected Tropical Diseases*	A028
C113	Hahn BH, Shaw GM, De KM, Cock, Sharp PM	*AIDS as a Zoonosis: Scientific and Public Health Implications*	10.1126/science.287.5453.607	*Science*	A028
C114	Gray RR, Tatem AJ, Lamers S, et al	*Spatial phylodynamics of HIV-1 epidemic emergence in east Africa*	10.1097/QAD.0b013e32832faf61	*AIDS*	A028
C115	Faria NR, Rambaut A, Suchard MA, et al.	*The early spread and epidemic ignition of HIV-1 in human populations*	10.1126/science.1256739	*Science*	A028
C116	Tomley FM, Shirley MW	*Livestock infectious diseases and zoonoses*	10.1098/rstb.2009.0133	*Philosophical Transactions of the Royal Society B: Biological Sciences*	A028
C117	Starr MD, Rojas JC, Zeledón R, Hird DW, Carpenter TE	*Chagas Disease: Risk Factors for House Infestation by Triatoma dimidiata, the Major Vector of Trypanosoma cruzi in Costa Rica*	10.1093/oxfordjournals.aje.a115949	*Proceedings of the National Academy of Sciences*	A028
C118	Schofield C, Diotaiuti L, Dujardin J.	*The process of domestication in triatominae*	10.1590/S0074-02761999000700073	*Memorias do Instituto Oswaldo Cruz*	A028
C119	Lima AFR, Jeraldo VDLS, Silveira MS, Madi RR, Santana TBK, Melo CMD	*Triatomines in dwellings and outbuildings in an endemic area of Chagas disease in northeastern Brazil*	10.1590/s0037-868220120000600009	*Revista da Sociedade Brasileira de Medicina Tropical*	A028

C120	Crocco L, Nattero J, López A, et al.	*Factors associated with the presence of triatomines in rural areas of south Argentine Chaco*	10.1590/0037-8682-0357-2018	*Revista da Sociedade Brasileira de Medicina Tropical*	A028
C121	Sokolow SH, Jones IJ, Jocque M, et al.	*Nearly 400 million people are at higher risk of schistosomiasis because dams block the migration of snail-eating river prawns*	10.1098/rstb.2016.0127	*Philosophical Transactions of the Royal Society B: Biological Sciences*	A028
C122	Hotez PJ	*Neglected Infections of Poverty among the Indigenous Peoples of the Arctic*	10.1371/journal.pntd.0000606	*PLoS Neglected Tropical Diseases*	A028
C123	Dos Santos RA, Osorio Severo D, Da Graça Luderitz Hoefel M	*Bolsonaro's hostility has driven Brazil's Indigenous peoples to the brink*	10.1038/d41586-020-02431-0	*Nature*	A028
C124	Dos Santos Costa AC, Ahmad S, Essar MY.	*Vaccination: Brazil fails Indigenous people again with two-tier scheme*	10.1038/d41586-021-01409-w	*Nature*	A028
C125	Hiraldo D, James K, Carroll SR	*Case Report: Indigenous Sovereignty in a Pandemic: Tribal Codes in the United States as Preparedness*	10.3389/fsoc.2021.617995	*Frontiers in Sociology*	A028
C126	Gracey M, King M	*Indigenous health part 1: determinants and disease patterns*	10.1016/S0140-6736(09)60914-4	*The Lancet*	A028
C127	Lau CL, Watson CH, Lowry JH, et al.	*Human Leptospirosis Infection in Fiji: An Eco-epidemiological Approach to Identifying Risk Factors and Environmental Drivers for Transmission*	10.1371/journal.pntd.0004405	*PLoS Neglected Tropical Diseases*	A028
C128	Bausch DG, Borchert M, Grein T, et al.	*Risk Factors for Marburg Hemorrhagic Fever, Democratic Republic of the Congo*	10.3201/eid0912.030355	*Emerging Infectious Diseases*	A028
C129	Pawęska JT, Jansen Van Vuren P, Kemp A, et al.	*Marburg Virus Infection in Egyptian Rousette Bats, South Africa, 2013–2014*	10.3201/eid2406.172165	*Emerging Infectious Diseases*	A028
C130	Amman BR, Bird BH, Bakarr IA, et al.	*Isolation of Angola-like Marburg virus from Egyptian rousette bats from West Africa*	10.1038/s41467-020-14327-8	*Nature Communications*	A028
